# Supplementary material for: Social and structural determinants of injecting-related bacterial and fungal infections among people who inject drugs: protocol for a mixed studies systematic review
Source: BMJ Open. 2021 Aug 9;11(8):e049924. doi: 10.1136/bmjopen-2021-049924 (PMC8354281; doi:10.1136/bmjopen-2021-049924)
Supplement: Supplementary data [file bmjopen-2021-049924supp001.pdf]

Supplementary materials for the article:

Social and structural determinants of injecting-related bacterial and fungal infections among people who inject drugs: protocol for a mixed studies systematic review

Thomas D. Brothers[1,2]; Dan Lewer[1]; Matthew Bonn[3]; Duncan Webster[2,4]; Magdalena Harris[5]

[1]UCL Collaborative Centre for Inclusion Health, Institute of Epidemiology & Health Care, University College London (UCL), London, UK
[2]Department of Medicine, Dalhousie University, Halifax, Canada
[3]Canadian Association of People who Use Drugs (CAPUD), Dartmouth, Canada
[4]Division of Infectious Diseases, Saint John Regional Hospital, Saint John, Canada
[5]Department of Public Health, Environments and Society, London School of Hygiene and Topical Medicine (LSHTM), London, UK

This material supplements, but does not replace, the peer-reviewed article in BMJ Open

Supplementary appendix Table 1. Pilot search strategy developed in PubMed on February 1, 2021 (1,752 results)

Concepts	PubMed
People who inject drugs, or drug preparation and injection	(“Substance-Related Disorders”[MeSH] OR
	“Substance Abuse, Intravenous”[MeSH] OR
	“Drug Users”[MeSH] OR
	“Needle Sharing”[MeSH] OR
	“people who inject drugs”[tiab] OR “persons who inject drugs”[tiab] OR PWID[tiab] OR
	“people who use drugs”[tiab] OR “persons who use drugs”[tiab] OR PWUD[tiab] OR
	“injection drug”[tiab] OR IDU[tiab] OR
	“intravenous drug”[tiab] OR IVDU[tiab] OR
	“drug abuse”[tiab] OR
	“illicit drugs”[MeSH] OR “illicit drug”[tiab] OR “illegal drug”[tiab] OR
	“Heroin”[MeSH] OR Heroin[tiab] OR
	“Heroin Dependence”[MeSH] OR
	“Opiate use disorder”[tiab] OR “opioid use disorder”[tiab] OR “opiate dependence”[tiab] OR “opioid dependence”[tiab] OR “opiate abuse”[tiab] OR “opioid abuse”[tiab] OR “substance use disorder”[tiab] OR “substance abuse”[tiab] OR
	“Cocaine”[MeSH] OR cocaine[tiab] OR
	“Crack Cocaine”[MeSH] OR
	“groin injecting”[tiab] OR “femoral injecting”[tiab] OR
	“Harm Reduction”[MeSH] OR “harm reduction”[tiab] OR
	“Needle-Exchange Programs”[MeSH] OR

	"needle exchange"[tiab] OR "syringe exchange"[tiab] OR "syringe services"[tiab] OR "syringe program"[tiab] OR
	acidifier*[tiab] OR
	"Opiate Substitution Treatment"[MeSH] OR "opiate substitution"[tiab] OR "opiate agonist"[tiab] OR "opioid substitution"[tiab] OR "opioid agonist"[tiab] OR
	"Medications for opioid use disorder"[tiab] OR MOUD[tiab] OR
	Methadone[tiab] OR
	Buprenorphine[tiab] OR
	Hydromorphone[tiab] OR morphine[tiab] or fentanyl[tiab])
Injecting-related infections	AND
	("injection-related infections"[tiab] OR "injection-related infection"[tiab] OR
	"bacterial infection"[tiab] OR "bacterial infections"[tiab] OR
	Bacteremia[MeSH] OR bacteremia[tiab] OR
	Fungemia[MeSH] OR
	Cellulitis[MeSH] OR cellulitis[tiab] OR
	Abscess[MeSH] OR abscess*[tiab] OR
	"skin infection"[tiab] OR "skin infections"[tiab] OR
	"skin and soft tissue"[tiab] OR SSTI*[tiab] OR
	Endocarditis[MeSH] OR endocarditis[tiab] OR
	Bone Diseases, Infectious[MeSH] OR
	Osteomyelitis[MeSH] OR Osteomyelitis[tiab] OR
	"septic arthritis"[tiab] OR
	Central Nervous System Infections[MeSH] OR

	Gram-Positive Bacterial Infections[MeSH] OR<br>Candidiasis[MeSH])
Social and structural determinants, or risk environment	AND
	("risk factor"[tiab] OR “risk factors”[tiab] OR<br>correlate*[tiab] OR<br>determinant*[tiab] OR<br>environment*[tiab] OR<br>“social factors”[tiab] or “structural factors”[tiab] OR<br>Cohort*[tiab] OR<br>Longitudinal[tiab] OR<br>Prospective[tiab] OR retrospective[tiab] OR<br>Randomized[tiab] OR randomised[tiab] OR<br>Comparative[tiab] OR<br>Case-control[tiab] OR<br>Time-series[tiab] OR<br>Survey*[tiab] OR<br>Epidemiolog*[tiab] OR<br>Qualitative[tiab] OR<br>Interview[tiab] OR<br>Ethnograph*[tiab] OR<br>Mixed-methods[tiab] OR “mixed methods”[tiab] OR<br>gender[tiab] OR<br>homeless*[tiab] OR

race[tiab] OR racism[tiab] OR

incarcerat*[tiab] OR prison*[tiab] OR criminal*[tiab] OR

stigma*[tiab] OR discrimination[tiab] OR exclusion[tiab])

NOT (“case report”[Title]) NOT (“case series”[Title])

Filter: 2000-Present
