# Supplementary material for: Social and structural determinants of injecting-related bacterial and fungal infections among people who inject drugs: protocol for a mixed studies systematic review
Source: BMJ Open. 2021 Aug 9;11(8):e049924. doi: 10.1136/bmjopen-2021-049924 (PMC8354281; doi:10.1136/bmjopen-2021-049924)
Supplement: Author's manuscript [file bmjopen-2021-049924.draft_revisions.pdf]

BMJ Open is committed to open peer review. As part of this commitment we make the peer review history of every article we publish publicly available.

When an article is published we post the peer reviewers' comments and the authors' responses online. We also post the versions of the paper that were used during peer review. These are the versions that the peer review comments apply to.

The versions of the paper that follow are the versions that were submitted during the peer review process. They are not the versions of record or the final published versions. They should not be cited or distributed as the published version of this manuscript.

BMJ Open is an open access journal and the full, final, typeset and author-corrected version of record of the manuscript is available on our site with no access controls, subscription charges or pay-per-view fees (http://bmjopen.bmj.com).

If you have any questions on BMJ Open's open peer review process please email info.bmjopen@bmj.com

BMJ Open

Social and structural determinants of injecting-related bacterial and fungal infections among people who inject drugs: protocol for a mixed studies systematic review

Journal:	*BMJ Open*
Manuscript ID	bmjopen-2021-049924
Article Type:	Protocol
Date Submitted by the Author:	04-Feb-2021
Complete List of Authors:	Brothers, Thomas; UCL Institute of Epidemiology and Health Care, UCL Collaborative Centre for Inclusion Health; Dalhousie University, Department of Medicine<br>Lewer, Dan; UCL Institute of Epidemiology and Health Care, UCL Collaborative Centre for Inclusion Health<br>Bonn, Matthew; Canadian Association of People Who Use Drugs, Dartmouth, Nova Scotia,<br>Webster, Duncan; Dalhousie University, Department of Medicine; Saint John Regional Hospital, Division of Infectious Diseases<br>Harris, Magdalena; London School of Hygiene & Tropical Medicine, Department of Public Health, Environments and Society
Keywords:	Substance misuse < PSYCHIATRY, Infectious diseases & infestations < DERMATOLOGY, INFECTIOUS DISEASES, SOCIAL MEDICINE, Public health < INFECTIOUS DISEASES, PUBLIC HEALTH

SCHOLARONE™
Manuscripts

I, the Submitting Author has the right to grant and does grant on behalf of all authors of the Work (as defined in the below author licence), an exclusive licence and/or a non-exclusive licence for contributions from authors who are: i) UK Crown employees; ii) where BMJ has agreed a CC-BY licence shall apply, and/or iii) in accordance with the terms applicable for US Federal Government officers or employees acting as part of their official duties; on a worldwide, perpetual, irrevocable, royalty-free basis to BMJ Publishing Group Ltd ("BMJ") its licensees and where the relevant Journal is co-owned by BMJ to the co-owners of the Journal, to publish the Work in this journal and any other BMJ products and to exploit all rights, as set out in our licence.

The Submitting Author accepts and understands that any supply made under these terms is made by BMJ to the Submitting Author unless you are acting as an employee on behalf of your employer or a postgraduate student of an affiliated institution which is paying any applicable article publishing charge ("APC") for Open Access articles. Where the Submitting Author wishes to make the Work available on an Open Access basis (and intends to pay the relevant APC), the terms of reuse of such Open Access shall be governed by a Creative Commons licence – details of these licences and which Creative Commons licence will apply to this Work are set out in our licence referred to above.

Other than as permitted in any relevant BMJ Author's Self Archiving Policies, I confirm this Work has not been accepted for publication elsewhere, is not being considered for publication elsewhere and does not duplicate material already published. I confirm all authors consent to publication of this Work and authorise the granting of this licence.

For peer review only

Social and structural determinants of injecting-related bacterial and fungal infections among people who inject drugs: protocol for a mixed studies systematic review

Thomas D. Brothers[1,2]; Dan Lewer[1]; Matthew Bonn[3]; Duncan Webster[2,4]; Magdalena Harris[5]

[1]UCL Collaborative Centre for Inclusion Health, Institute of Epidemiology & Health Care, University College London (UCL), London, UK
[2]Department of Medicine, Dalhousie University, Halifax, Canada
[3]Canadian Association of People who Use Drugs (CAPUD), Dartmouth, Canada
[4]Division of Infectious Diseases, Saint John Regional Hospital, Saint John, Canada
[5]Department of Public Health, Environments and Society, London School of Hygiene and Topical Medicine (LSHTM), London, UK

Address correspondence to:
Thomas D. Brothers, MD
483 Bethune Building
1276 South Park St.
Halifax, NS B3H 2Y9
thomas.brothers@dal.ca

Word count: 2942

Email & ORCiD:
Thomas D. Brothers: thomas.brothers@dal.ca; 0000-0002-5570-5556
Dan Lewer: d.lewer@ucl.ac.uk; 0000-0003-3698-7196
Matthew Bonn: matthewbonn00@gmail.com; 0000-0002-6406-0171
Duncan Webster: duncan.webster@horizonnb.ca; 0000-0001-7692-7150
Magdalena Harris: magdalena.harris@lshtm.ac.uk; 0000-0001-8718-8226

For peer review only

ABSTRACT

Introduction: Injecting-related bacterial and fungal infections are a common complication among people who inject drugs (PWID), associated with significant morbidity and mortality. Invasive infections, including infective endocarditis, appear to be increasing in incidence. To date, preventive efforts have focused on modifying individual-level risk behaviours (e.g. handwashing, skin cleaning) without much success in reducing the population-level impact of these infections. Learning from successes in HIV prevention, there may be great value in looking beyond individual-level risk behaviours to the social determinants of health. Specifically, the risk environment conceptual framework identifies how social, physical, economic, and political environmental factors facilitate and constrain individual behaviour, and therefore influence health outcomes. Understanding the social and structural determinants of injecting-related bacterial and fungal infections could help to identify new targets for prevention efforts in the face of increasing incidence of severe disease.

Methods and analysis: This is a protocol for a systematic review. We will review studies of PWID and investigate associations between risk factors (both individual-level and social/structural-level) and the incidence of hospitalization or death due to injecting-related bacterial infections (skin and soft-tissue infections, bacteremia, infective endocarditis, osteomyelitis, septic arthritis, epidural abscess, and others). We will include quantitative, qualitative, and mixed methods studies. Using directed content analysis, we will code risk factors for these infection-related outcomes according to their contributions to the risk environment in type (social, physical, economic, or political) and level (micro- or macro-environmental). We will also code and present risk factors at each stage in the process of drug acquisition, preparation, injection, superficial infection care, severe infection care or hospitalization, and outcomes after infection or hospital discharge.

For peer review only - http://bmjopen.bmj.com/site/about/guidelines.xhtml

Ethics and dissemination: As an analysis of published literature, no ethics approval is required. The findings will inform a research agenda to develop and implement social/structural interventions aimed at reducing the burden of disease.

PROSPERO registration number: CRD42021231411

Keywords: injection drug use; injecting drug use; cellulitis; abscess; endocarditis; social determinants; risk environment

For peer review only

BOX

Strengths and limitations of this study

- Injecting-related bacterial and fungal infections are a common problem among people who inject drugs (PWID), associated with significant morbidity and mortality.
- Understanding the social and structural determinants of injecting-related bacterial and fungal infections could help to identify new targets for prevention efforts, in the face of increasing incidence of severe disease.
- People with lived and living expertise of injection drug use, as well as practicing clinicians caring for PWID with injecting-related infections and experienced qualitative, quantitative, and mixed methods researchers, were involved in the development of the protocol and will participate in analysis and synthesis.
- Reviewing quantitative, qualitative, and mixed methods studies enables a broad scope and the inclusion of important contextual information that may not be captured in quantitative studies alone.
- This review only includes studies with infection-related outcomes, and so may not include some studies with risk factors for infection (e.g. hand-washing, skin-cleaning) as outcomes.

For peer review only

INTRODUCTION

Injecting-related bacterial infections are very common among people who inject drugs (PWID), with up to 39% of PWID reporting skin infections within the past month and 70% reported lifetime prevalence.[1] If not effectively treated, superficial infections can progress and spread through the bloodstream, leading to life-threatening infections such as infective endocarditis, osteomyelitis, septic arthritis, or epidural abscess.[2] Pathogenic bacteria or fungi may be introduced directly into the bloodstream as endogenous skin flora, or from non-sterile injecting equipment or contaminated drugs.[3] As a result, PWID face extremely high risk of disability or death from severe bacterial and fungal infections that require hospitalization for antibiotics and/or surgery.[4–9] The incidence of severe bacterial and fungal infections is increasing among PWID in Canada[10–12], the United Kingdom [13], the USA [14–19], Australia [20], and Sweden.[21] A recent modelling study suggests that one in five PWID in the USA may die of infective endocarditis alone over the next 10 years.[19] Better approaches to prevention are urgently needed.

To date, prevention efforts for injecting-related bacterial and fungal infections have primarily focused on changing individual-level risk behaviours,[1,22] including hand-washing before drug preparation,[23] skin-cleaning before injecting,[24] and avoiding subcutaneous or intramuscular injections.[25] While individual-level interventions may be effective for people who due to their socioeconomic situation can adopt these practices,[26,27] they have not translated into reductions in the population-level incidence of severe bacterial and fungal infections, which continue to rise.

An appreciation for the importance of social and structural determinants of health has emerged from the understanding that individual health behaviours are shaped by contextual factors.[28–32] The "risk environment" conceptual framework, as developed by Rhodes and others,[33–36] describes how

For peer review only - http://bmjopen.bmj.com/site/about/guidelines.xhtml

interactions between social and structural factors external to the individual influence individual behaviours, and therefore structure or create health harms.[37] The risk environment framework has informed clinical and public health efforts at reducing other drug-related harms, including human immunodeficiency virus (HIV) transmission,[33,36,38] hepatitis C virus (HCV) treatment,[39] and non-fatal and fatal overdoses.[29,40] Better understanding of the social determinants of health can help to shift beliefs about responsibility and risk from individual behaviours to the places and social situations in which individuals exist, and inform the development of innovative interventions addressing both social and individual-level factors.[36,41]

Like other drug-related harms, risk for injecting-related bacterial and fungal infections also likely reflects contributions of multiple, interacting factors external to individuals that influence risk behaviours and therefore shape health outcomes. For example, homelessness may constrain an individual's ability to wash their hands or use sterile water for injecting,[42] and policy constraints on needle and syringe programs (NSP) (from criminalisation to reduced operating hours) create a situation in which an individual is more likely to reuse a blunted or contaminated needle. In response to the alarming increases in incidence, calls to enhance understanding of the social determinants of injecting-related bacterial and fungal infections have emerged from both PWID[43] and academic communities.[2,4,26,44] Learning from the successes of HIV prevention efforts in particular, mapping the risk environment for injecting-related bacterial and fungal infections could inform new prevention efforts that target social and structural causes.[45,46]

Objectives

In order to better understand risk for injecting-related bacterial and fungal infections among PWID, we seek to conduct a systematic mixed studies review (of quantitative, qualitative, and mixed methods

For peer review only - http://bmjopen.bmj.com/site/about/guidelines.xhtml

evidence) to identify social and structural determinants of these infections among PWID, informed by the risk environment framework. The proposed review will seek to answer the question, “Among people who inject drugs (PWID), what social and structural factors are associated with the development, treatment, and outcomes of injecting-related bacterial and fungal infections?”

METHODS AND ANALYSIS

This systematic review protocol follows the Preferred Reporting Items for Systematic Review and Meta-Analysis Protocols (PRISMA-P) guideline[47,48] and is informed by guidance on conducting systematic reviews of association (etiology) by the Joanna Briggs Institute[49] and on conducting and reporting mixed studies reviews by Pluye and colleagues[50,51] and by Tricco and colleagues.[52]

“Mixed studies reviews” incorporate synthesis of quantitative, qualitative, and mixed methods studies; they are one of several emerging methods for systematically incorporating evidence from diverse methodologies (e.g. integrative review, realist review, meta-narrative review, critical interpretive synthesis).[50,52,53] Mixed studies reviews use a mixed methods approach.[54,55] They are particularly useful for research questions like ours that require detailed contextual information available from qualitative and mixed methods studies, and for synthesis plans like ours that incorporate a qualitative content analysis directed by existing theories and conceptual frameworks.[50,52]

This systematic mixed studies review protocol is registered with PROSPERO (registration number CRD42021231411). We have searched the PROSPERO registry of systematic reviews and found none in progress related to our objectives.

Conceptual framework

For peer review only

The risk environment framework is the most prominent social-ecological model for substance use research; it comprises risk factors external to individuals, considering types and levels of environmental influence.[34,35,37,56] As first developed in the context of HIV prevention, the risk environment framework describes four different types of environmental influences: social, physical, economic, and policy. These can occur at two different levels, microenvironmental or macroenvironmental.[38] Microenvironmental factors operate at the level of interpersonal relationships, community and group norms, and institutional or organizational responses.[36] This could include local norms about the culture of substance use and acceptability of receptive sharing or reuse of potentially contaminated injecting equipment among PWID (a social factor), or increasing housing prices (an economic factor) contributing to homelessness and lack of access to soap and water (physical factors). Macroenvironmental factors operate at the level of states, societies, and laws, and interact with microenvironmental factors.[36] This could include state policing crackdowns on heroin importation leading instead to increased importation of fentanyl (a policy factor), which has a shorter half-life and associated risks of increased injecting frequency.[57]

Eligibility criteria

Informed by the Population, Exposures, Outcomes (PEO) approach,[49] studies will be selected according to the following criteria.

Study designs

We will include quantitative, qualitative, and mixed methods peer-reviewed studies assessing factors associated with the outcomes of interest (i.e., health outcomes related to injecting-related bacterial and fungal infections, as described below). We will therefore exclude case-reports, case series, reviews, commentaries, and editorials, as they do not include analyses of association.

For peer review only

Participants

We will include studies examining PWID of any age or nationality. By PWID, we are referring to people who inject opioids (e.g. heroin, fentanyl, hydromorphone, morphine), stimulants (e.g. cocaine, methamphetamines), or other psychoactive substances via intravenous, intramuscular, or subcutaneous routes. We expect there to be differences between studies in how PWID are operationally defined (e.g. ever injected, recently injected in past year or past 3 months, etc.).[1] Taking a broad perspective, we will include studies that identify participants as PWID at risk for injecting-related infections, and not limit this to self-reported current or recent injecting. Many people who inject drugs transition back and forth between injection and non-injection routes of consumption,[58,59] and PWID who have stopped injecting may experience persistently increased risk for invasive infections such as endocarditis, due to prior damage to vasculature and heart valves.[2] Studies that focus only on people who inject performance-enhancing drugs (e.g. anabolic steroids) or gender-affirming hormones will be excluded as these are not psychoactive substances.

Exposures of interest

We will include studies addressing risk factors or determinants for injecting-related bacterial and fungal infections among PWID, taking a broad perspective. In addition to studies that directly survey individual participants about potential risk factors, we will consider studies investigating potential associations with social or structural risk factors at micro-environmental and macro-environmental levels. We will also include studies of interventions that aim to reduce risk, including educational, behavioural, or policy interventions. Studies that only report on individual-level risk factors (e.g. skin-cleaning) will be included in the search, and then classified separately in the data synthesis.

For peer review only - http://bmjopen.bmj.com/site/about/guidelines.xhtml

Outcomes

Outcomes of interest include incidence of, hospitalization with, or mortality due to, injecting-related bacterial and fungal infections. Bacterial and fungal infections include skin and soft-tissue infections (cellulitis, abscess, necrotizing fasciitis), bloodstream infections (bacteremia), vascular infections (endocarditis, septic or suppurative phlebitis), bone and joint infections (osteomyelitis, septic arthritis, discitis), and central nervous system infections (epidural abscess, brain abscess, meningitis, encephalitis). We will also include as outcomes, diseases caused by pathogenic bacteria and fungi associated with drug supply, preparation, and injecting practices, including tetanus,[22] botulism[3], invasive group A streptococcal disease,[60] as well as methicillin-sensitive *Staphylococcus aureus* (MSSA) and methicillin-resistant *Staphylococcus aureus* (MRSA).[61] We will exclude studies identifying only non-injecting related bacterial and fungal infections as outcomes, such as tuberculosis, pneumonia, chlamydia, or gonorrhea, and bacterial infections that are primarily sexually transmitted, e.g. syphilis.

We will extract all composite and individual outcomes as reported. We expect there to be many different methods for identifying these outcomes, including participant self-report of infection or hospitalization, administrative records of hospital admission, and mortality records from vital statistics.

Time frame, setting, and language

We will include studies from 2000 onward, to capture contemporary research that would be more likely to inform policy and clinical practice. There will be no restrictions by study setting. We will include articles in English and French. A list of potentially relevant titles reported in other languages will be provided as an appendix.

Information sources

For peer review only - http://bmjopen.bmj.com/site/about/guidelines.xhtml

For peer review only

We will search PubMed, EMBASE, Scopus, CINAHL, and PsycINFO databases. Electronic database searches will be supplemented by manually reviewing reference lists of included studies, and forward "snowball" searching by identifying articles that cite included studies.[62] We will also circulate a bibliography of the included articles to the systematic review team, which includes people with lived (past) and living (current) expertise of injection drug use and clinicians who care for people with injection-associated infections. We will include articles identified from the personal files of the systematic review team that were not identified in the bibliography.

Search strategy

Our search strategy is informed by several key existing systematic reviews or review protocols. A 2017 systematic review identified prevalence and individual-level risk factors for injecting-related injuries and diseases among PWID, including bacterial infections.[1] Previous systematic reviews have applied the risk environment framework to understand HIV transmission[33], HIV[63] and HCV[39] treatment access, HIV disease progression,[64] and "safer environment interventions"[41] among PWID. Two published protocols of systematic reviews in progress focused on health services and interventions to treat or prevent viral and bacterial infections among PWID.[65,66]

The final search strategy will be developed by our review team in consultation with health information specialist-librarians with systematic review experience. We conducted a pilot search in PubMed and validated by checking for inclusion of key, recent studies known to the authors that assessed either risk factors for severe bacterial or fungal infections among PWID,[67,68] social/structural determinants of bacterial infections among PWID,[42,69–72] or complex interventions to reduce risk of bacterial infections among PWID[26] (Appendix Table 1). This validation process led to several iterative changes,

For peer review only - http://bmjopen.bmj.com/site/about/guidelines.xhtml

including specific search terms related to “acidifiers” and “groin injecting”. See Table 1 for the draft search strategy in PubMed, which identified 1,752 potentially relevant sources on February 1, 2021.

Data management and reference selection

Search results will be uploaded into Covidence, a cloud-based software program, where they can be automatically checked for de-duplication. Two reviewers will screen titles and abstracts against the inclusion criteria. We will obtain full text reports for all titles that appear to meet the inclusion criteria, or for where there is uncertainty. Two reviewers will screen these full text reports and mark each as included or excluded, recording reasons for exclusion. We will resolve discrepancies through discussion.

Data collection process

The team will develop and pilot-test a data extraction form, which can be applied in Covidence. We will extract information on:

- Study date (or publication date, if study date unavailable)
- Study country/region
- Methodology (quantitative, qualitative, or mixed methods)
- Study design
- Sample size and demographic characteristics (age, gender, race/ethnicity)
- Inclusion criteria and definition of PWID
- Recruitment setting (e.g. community recruitment, needle exchange clients, clinic, hospital, etc.)
- Outcomes reported (incident infection/diagnosis, clinic visit, emergency department visit, hospital admission, or death)
- Outcome operational criteria (self-reported, medical record review, administrative data)

For peer review only

- Infections reported (skin and soft-tissue infections at injection sites [e.g. abscess, cellulitis], bacteremia, infective endocarditis, osteomyelitis, septic arthritis, or other)[1]
- Risk factors assessed (including operational criteria)
- Measures of association between risk factors and outcomes (for quantitative and mixed methods studies)
- Summary of findings
- Implications for policy and/or practice, as reported by authors[65]
- Implications for research, as reported
- Gaps identified, as reported

Critical appraisal/Risk of bias assessment

We will apply a formal, validated critical appraisal tool for mixed studies reviews, the Mixed Methods Appraisal Tool (MMAT), 2018 edition, which is designed for use with quantitative, qualitative, and mixed methods studies.[73,73,74] It includes five core quality criteria for five categories of study designs: (a) qualitative, (b) randomized controlled/interventional, (c) nonrandomized controlled, (d) quantitative descriptive, and (e) mixed methods. This tool does not assign an overall quality score; rather, we will consider each item in the risk of bias assessment independently. Due to the broad scope of this review, we will not exclude studies deemed low quality. Study quality and risk of bias will inform interpretation of the data.

Data synthesis and analysis

We will perform a quantitative tabular synthesis of included studies, by year; country/region; methodology (quantitative, qualitative, or mixed methods), infection type (cellulitis, endocarditis, etc.);

For peer review only - http://bmjopen.bmj.com/site/about/guidelines.xhtml

and outcome (incident infection, care of superficial infection, care of severe infection/hospitalization, or outcomes after infection).

We will then conduct a qualitative, directed content analysis, as described by Hsieh and Shannon,[75] to map the risk environment for injecting-related bacterial and fungal infections. Using NVivo, we will first code each included exposure or risk factor according to the risk environment framework as individual-level or social/structural. Social/structural factors are those that are external to the individual, and together make up the risk environment. We will then code each social/structural factor according to the type (social, economic, political, or physical) and level (macro-environmental or micro-environmental) of the risk environment. We will use a hybrid deductive-inductive approach, as described by Pluye and Hong;[50] we will start with the existing risk environment conceptual framework but allow for new concepts related to the classification of specific factors to emerge. To improve reliability, coding will be performed in tandem by a reviewer pair for three studies. Coding will then be discussed with the whole review team to identify improvements to the coding approach and framework. The remaining studies will be coded by one reviewer. We will present a tabular synthesis categorizing each social/structural factor according to type and level in the risk environment framework,[49] and a narrative synthesis of the existing evidence for each social/structural factor.[39] See Table 1 for the model table framework.

For peer review only

In order to explore how different macro- and micro-environmental factors influence individual-level risk behaviours, we will then present a narrative synthesis of identified individual-level and social/structural factors affecting each step of a potential pathway from (a) drug acquisition; (b) drug preparation; (c) drug injection; (d) development of and care for superficial infections (e.g. self-treatment or primary/emergency department care); (e) development of and care for severe or invasive infections (e.g. self-care or hospital care); and (f) outcomes after infection or hospital discharge, including

For peer review only - http://bmjopen.bmj.com/site/about/guidelines.xhtml

infection-related mortality.[2] See Figure 1 and Figure 2 for illustrative schematics of how macro- and micro-environmental factors influence individual-level factors and may increase risks of injecting-related bacterial infections at two different stages in the pathway from drug acquisition to outcomes after severe/invasive infections. We plan to develop and refine these figures to reflect findings of the review. Note that because this review includes only studies with infection-related outcomes, we may not identify or include all published studies that identify determinants of intermediate risk factors or behaviours; for example some studies identifying determinants of skin cleaning before injecting may not be included in this review if they do not consider infection-related outcomes.

Patient and public involvement

The research team includes people with lived/living expertise of injection drug use and clinicians caring for PWID with bacterial and fungal injections. The research questions and analysis approach have been designed collaboratively, and build on existing relationships between authors with lived/living expertise and academic/medical expertise.[76–78] The topic was inspired by the deaths of friends and patients to injecting-related bacterial infections, and a recognition of the need to look broader than individual-level interventions.[79–81]

Ethics and dissemination

As this is a secondary analysis of published literature, no ethics approval is required. By mapping the risk environment for injecting-related bacterial and fungal infections among PWID, this study aims to identify opportunities for new policy and practice approaches to prevention. This may include highlighting the importance of scaling up access to existing interventions (e.g. needle/syringe distribution), identifying opportunities for novel combined social and clinical interventions (e.g. providing Housing First along with antibiotics and addiction treatment for PWID hospitalized with

For peer review only

invasive infections), or the potential for policy change (e.g. decriminalizing substance use may combat stigma and facilitate access to traditional primary care of injecting-related infections). The risk environment approach may also help to shift beliefs regarding responsibility and risk from individual behaviours to social and structural causes, which may change attitudes of health care professionals and systems regarding treatment of severe infections, including procedures such as heart valve replacement surgeries.[82,83] This may also inform educational and behaviour-change interventions, highlighting the importance of addressing more immediate, pragmatic priorities (e.g. housing and income, or vein access and care) that may also have benefits in infection risk reduction.[84] By emphasizing the potential importance of social and structural factors, the findings will also inform future epidemiological research using large-scale linked administrative data linking individual and clinical risk factors with social informatics.[68,85] Knowledge translation activities will include peer-reviewed academic publications and conference presentations, meetings with drug user groups/unions and harm reduction programs, and also communications to the lay public in targeted outlets.

For peer review only

Authors contributions

This study was conceived by TDB, DL, MB, and MH. The pilot search strategy was developed by TDB, and revised with input from DL and MD. TDB, DL, and MB will be involved in the data collection for this study. All authors will be involved in data interpretation and analysis. TDB wrote the first draft of this manuscript. All authors provided critical feedback and intellectual input. All authors provided their final approval for the publication of this version of the manuscript and agree to be accountable for all aspects of this work.

Funding statement: TDB is supported by the Dalhousie University Internal Medicine Research Foundation Fellowship, Killam Postgraduate Scholarship, Ross Stewart Smith Memorial Fellowship in Medical Research, and Clinician Investigator Program Graduate Stipend (all from Dalhousie University Faculty of Medicine), a Canadian Institutes of Health Research Fellowship [CIHR-FRN# 171259], and through the Research in Addiction Medicine Scholars (RAMS) Program (National Institutes of Health/National Institute on Drug Abuse; R25DA033211). DL is funded by a National Institute of Health Research Doctoral Research Fellowship [DRF-2018-11-ST2-016]. MB was supported in this work via the Ross Stewart Smith Memorial Fellowship in Medical Research, from Dalhousie University Faculty of Medicine. MH is funded by a National Institute of Health Research Career Development Fellowship [CDF-2016-09-014]. The views expressed are those of the author(s) and not necessarily those of the NHS, the NIHR or the Department of Health and Social Care. These funders had no role in the conduct or reporting of the research.

Declaration of interests

For peer review only

MB reports personal fees from AbbVie, a pharmaceutical research and development company, and grants and personal fees from Gilead Sciences, a research-based biopharmaceutical company, outside of the submitted work. The other authors have no conflicts to declare.

Acknowledgements

We appreciate the contributions to science of all participants in the included studies. TDB, MB, and DW live and work in Mi'kma'ki and along the Wolastoq, the ancestral and unceded territory of the Mi'kmaq and the Wolastoqiyik. We are all treaty people.

For peer review only

For peer review only - http://bmjopen.bmj.com/site/about/guidelines.xhtml

REFERENCES

1 Larney S, Peacock A, Mathers BM, *et al.* A systematic review of injecting-related injury and disease among people who inject drugs. *Drug Alcohol Depend* 2017;**171**:39–49. doi:10.1016/j.drugalcdep.2016.11.029

2 Robertson R, Broers B, Harris M. Injecting drug use, the skin and vasculature. *Addiction* 2020;:add.15283. doi:10.1111/add.15283

3 Trayner KMA, Weir A, McAuley A, *et al.* A pragmatic harm reduction approach to manage a large outbreak of wound botulism in people who inject drugs, Scotland 2015. *Harm Reduct J* 2018;**15**:36. doi:10.1186/s12954-018-0243-9

4 Wurcel AG. Drug-Associated Infective Endocarditis Trends: What's All the Buzz About? *Ann Intern Med* 2019;**170**:68. doi:10.7326/M18-3026

5 Oh DHW, Wurcel AG, Tybor DJ, *et al.* Increased Mortality and Reoperation Rates After Treatment for Septic Arthritis of the Knee in People Who Inject Drugs: Nationwide Inpatient Sample, 2000-2013. *Clin Orthop Relat Res* 2018;**476**:1557–65. doi:10.1097/01.blo.0000534682.68856.d8

6 Serota DP, Barocas JA, Springer SA. Infectious complications of addiction: A call for a new subspecialty within infectious diseases. *Clin Infect Dis* 2020;**70**:968–72. doi:10.1093/cid/ciz804

7 Lewer D, Freer J, King E, *et al.* Frequency of healthcare utilisation by adults who use illicit drugs: a systematic review and meta-analysis. *Addiction* 2020;**115**:1011–23. doi:10.1111/add.14892

8 Lewer Dan, Hope VD, Harris Magdalena, *et al.* Incidence and treatment costs of severe bacterial infections among people who inject heroin: A cohort study in South London, England. *Drug and Alcohol Dependence* 2020;**212**:108057. doi:10.1016/j.drugalcdep.2020.108057

9 Wright T, Hope V, Ciccarone D, *et al.* Prevalence and severity of abscesses and cellulitis, and their associations with other health outcomes, in a community-based study of people who inject drugs in London, UK. *PLOS ONE* 2020;**15**:e0235350. doi:10.1371/journal.pone.0235350

10 Weir MA, Slater J, Jandoc R, *et al.* The risk of infective endocarditis among people who inject drugs: a retrospective, population-based time series analysis. *CMAJ* 2019;**191**:E93–9. doi:10.1503/cmaj.180694

11 Coverdale N, Brogly S, Payne D, *et al.* Rates of Infective Endocarditis in Substance Use Disorder and Associated Costs in Ontario. *Can J Addict* 2019;**10**:36–43. doi:10.1097/CXA.0000000000000053

12 Mosseler K, Materniak S, Brothers TD, *et al.* Epidemiology, microbiology, and clinical outcomes among patients with intravenous drug use-associated infective endocarditis in New Brunswick. *CJC Open* 2020;**2**:379–85. doi:https://doi.org/10.1016/j.cjco.2020.05.002

13 Lewer D, Harris M, Hope V. Opiate Injection–Associated Skin, Soft Tissue, and Vascular Infections, England, UK, 1997–2016. *Emerging Infectious Diseases* 2019;**23**:1400–3. doi:10.3201/eid2308.170439

For peer review only

For peer review only - http://bmjopen.bmj.com/site/about/guidelines.xhtml

14 Bates MC, Annie F, Jha A, *et al.* Increasing incidence of IV-drug use associated endocarditis in southern West Virginia and potential economic impact. *Clin Cardiol* 2019;**42**:432–7. doi:10.1002/clc.23162

15 Deo SV, Raza S, Kalra A, *et al.* Admissions for Infective Endocarditis in Intravenous Drug Users. *J Am Coll Cardiol* 2018;**71**:1596–7. doi:10.1016/j.jacc.2018.02.011

16 Fleischauer AT. Hospitalizations for Endocarditis and Associated Health Care Costs Among Persons with Diagnosed Drug Dependence — North Carolina, 2010–2015. *MMWR Morb Mortal Wkly Rep* 2017;**66**. doi:10.15585/mmwr.mm6622a1

17 Schranz AJ, Fleischauer A, Chu VH, *et al.* Trends in Drug Use–Associated Infective Endocarditis and Heart Valve Surgery, 2007 to 2017: A Study of Statewide Discharge Data. *Ann Intern Med* 2019;**170**:31. doi:10.7326/M18-2124

18 Wurcel AG, Anderson JE, Chui KKH, *et al.* Increasing Infectious Endocarditis Admissions Among Young People Who Inject Drugs. *Open Forum Infect Dis* 2016;**3**. doi:10.1093/ofid/ofw157

19 Barocas JA, Eftekhari Yazdi G, Savinkina A, *et al.* Long-term Infective Endocarditis Mortality Associated With Injection Opioid Use in the United States: A Modeling Study. *Clin Infect Dis* 2020;**Published ahead of print.** doi:10.1093/cid/ciaa1346

20 Wright A, Otome O, Harvey C, *et al.* The Current Epidemiology of Injecting Drug Use-Associated Infective Endocarditis in Victoria, Australia in the Midst of Increasing Crystal Methamphetamine Use. *Heart Lung Circ* 2018;**27**:484–8. doi:10.1016/j.hlc.2017.03.166

21 Asgeirsson H, Thalme A, Weiland O. Low mortality but increasing incidence of Staphylococcus aureus endocarditis in people who inject drugs. *Medicine (Baltimore)* 2016;**95**. doi:10.1097/MD.0000000000005617

22 Gordon RJ, Lowy FD. Bacterial Infections in Drug Users. *New England Journal of Medicine* 2005;**353**:1945–54. doi:10.1056/NEJMra042823

23 Dwyer R, Topp L, Maher L, *et al.* Prevalences and correlates of non-viral injecting-related injuries and diseases in a convenience sample of Australian injecting drug users. *Drug and Alcohol Dependence* 2009;**100**:9–16. doi:10.1016/j.drugalcdep.2008.08.016

24 Hope VD, Hickman M, Parry JV, *et al.* Factors associated with recent symptoms of an injection site infection or injury among people who inject drugs in three English cities. *Int J Drug Policy* 2014;**25**:303–7. doi:10.1016/j.drugpo.2013.11.012

25 Phillips KT, Stein MD. Risk Practices Associated with Bacterial Infections among Injection Drug Users in Denver, Colorado. *Am J Drug Alcohol Abuse* 2010;**36**:92–7. doi:10.3109/00952991003592311

26 Roux P, Donadille C, Magen C, *et al.* Implementation and evaluation of an educational intervention for safer injection in people who inject drugs in Europe: a multi-country mixed-methods study. *Int J Drug Policy* 2021;**87**:102992. doi:10.1016/j.drugpo.2020.102992

For peer review only

For peer review only - http://bmjopen.bmj.com/site/about/guidelines.xhtml

27 Stein MD, Phillips KT, Herman DS, *et al.* Skin-cleaning among hospitalized people who inject drugs: a randomized controlled trial. *Addiction* 2020;**Online ahead of print**. doi:https://doi.org/10.1111/add.15236

28 Bourgois P, Holmes SM, Sue K, *et al.* Structural Vulnerability: Operationalizing the Concept to Address Health Disparities in Clinical Care. *Acad Med* 2017;**92**:299–307. doi:10.1097/ACM.0000000000001294

29 Dasgupta N, Beletsky L, Ciccarone D. Opioid Crisis: No Easy Fix to Its Social and Economic Determinants. *Am J Public Health* 2017;**108**:182–6. doi:10.2105/AJPH.2017.304187

30 Okechukwu C, Davison K, Emmons K. Changing Health Behaviors in a Social Context. In: Berkman LF, Kawachi I, Glymour MM, eds. *Social Epidemiology*. Oxford University Press 2014. 365–95. doi:10.1093/med/9780195377903.003.0010

31 Glass TA, McAtee MJ. Behavioral science at the crossroads in public health: Extending horizons, envisioning the future. *Soc Sci Med* 2006;**62**:1650–71. doi:10.1016/j.socscimed.2005.08.044

32 Luchenski S, Maguire N, Aldridge RW, *et al.* What works in inclusion health: overview of effective interventions for marginalised and excluded populations. *Lancet* 2018;**391**:266–80. doi:10.1016/S0140-6736(17)31959-1

33 Strathdee SA, Hallett TB, Bobrova N, *et al.* HIV and risk environment for injecting drug users: the past, present, and future. *Lancet* 2010;**376**:268–84. doi:10.1016/S0140-6736(10)60743-X

34 Rhodes T. The ‘risk environment’: a framework for understanding and reducing drug-related harm. *International Journal of Drug Policy* 2002;**13**:85–94. doi:10.1016/S0955-3959(02)00007-5

35 Rhodes T. Risk environments and drug harms: A social science for harm reduction approach. *Int J Drug Policy* 2009;**20**:193–201. doi:10.1016/j.drugpo.2008.10.003

36 Rhodes T, Singer M, Bourgois P, *et al.* The social structural production of HIV risk among injecting drug users. *Soc Sci Med* 2005;**61**:1026–44. doi:10.1016/j.socscimed.2004.12.024

37 Collins AB, Boyd J, Cooper HLF, *et al.* The intersectional risk environment of people who use drugs. *Soc Sci Med* 2019;**234**:112384. doi:10.1016/j.socscimed.2019.112384

38 Rhodes T, Simic M. Transition and the HIV risk environment. *BMJ* 2005;**331**:220–3.

39 Harris M, Rhodes T. Hepatitis C treatment access and uptake for people who inject drugs: a review mapping the role of social factors. *Harm Reduct J* 2013;**10**:7. doi:10.1186/1477-7517-10-7

40 McLean K. “There’s nothing here”: Deindustrialization as risk environment for overdose. *Int J Drug Policy* 2016;**29**:19–26. doi:10.1016/j.drugpo.2016.01.009

41 McNeil R, Small W. ‘Safer environment interventions’: A qualitative synthesis of the experiences and perceptions of people who inject drugs. *Soc Sci Med* 2014;**106**:151–8. doi:10.1016/j.socscimed.2014.01.051

For peer review only

42 Harris M, Scott J, Hope V, *et al.* Navigating environmental constraints to injection preparation: the use of saliva and other alternatives to sterile water among unstably housed PWID in London. *Harm Reduct J* 2020;**17**:24. doi:10.1186/s12954-020-00369-0

43 Unsigned. Heart Infection Projected to Kill One in Five People Who Inject Opioids by 2030. *Filter* 2020.https://filtermag.org/opioids-endocarditis-deaths/ (accessed 9 Dec 2020).

44 Harris M. Normalised pain and severe health care delay among people who inject drugs in London: Adapting cultural safety principles to promote care. *Social Science & Medicine* 2020;**260**:113183. doi:10.1016/j.socscimed.2020.113183

45 Perlman DC, Jordan AE. The Syndemic of Opioid Misuse, Overdose, HCV, and HIV: Structural-Level Causes and Interventions. *Curr HIV/AIDS Rep* 2018;**15**:96–112. doi:10.1007/s11904-018-0390-3

46 Blankenship KM, Friedman SR, Dworkin S, *et al.* Structural Interventions: Concepts, Challenges and Opportunities for Research. *J Urban Health* 2006;**83**:59–72. doi:10.1007/s11524-005-9007-4

47 Moher D, Shamseer L, Clarke M, *et al.* Preferred reporting items for systematic review and meta-analysis protocols (PRISMA-P) 2015 statement. *Syst Rev* 2015;**4**:1. doi:10.1186/2046-4053-4-1

48 Shamseer L, Moher D, Clarke M, *et al.* Preferred reporting items for systematic review and meta-analysis protocols (PRISMA-P) 2015: elaboration and explanation. *BMJ* 2015;**349**:g7647. doi:10.1136/bmj.g7647

49 Moola S, Munn Z, Sears K, *et al.* Conducting systematic reviews of association (etiology): The Joanna Briggs Institute's approach. *JBI Evidence Implementation* 2015;**13**:163–9. doi:10.1097/XEB.0000000000000064

50 Pluye P, Hong QN. Combining the Power of Stories and the Power of Numbers: Mixed Methods Research and Mixed Studies Reviews. *Annu Rev Public Health* 2014;**35**:29–45. doi:10.1146/annurev-publhealth-032013-182440

51 Pluye P, Hong QN, Bush PL, *et al.* Opening-up the definition of systematic literature review: the plurality of worldviews, methodologies and methods for reviews and syntheses. *J Clin Epidemiol* 2016;**73**:2–5. doi:10.1016/j.jclinepi.2015.08.033

52 Tricco AC, Antony J, Soobiah C, *et al.* Knowledge synthesis methods for integrating qualitative and quantitative data: a scoping review reveals poor operationalization of the methodological steps. *J Clin Epidemiol* 2016;**73**:29–35. doi:10.1016/j.jclinepi.2015.12.011

53 Dixon-Woods M, Agarwal S, Jones D, *et al.* Synthesising qualitative and quantitative evidence: A review of possible methods. *J Health Serv Res Policy* 2005;**10**:45–53. doi:10.1177/135581960501000110

54 Heyvaert M, Hannes K, Onghena P. *Using Mixed Methods Research Synthesis for Literature Reviews: The Mixed Methods Research Synthesis Approach*. SAGE Publications 2016.

55 Sandelowski M, Leeman J, Knafl K, *et al.* Text-in-context: a method for extracting findings in mixed-methods mixed research synthesis studies. *J Adv Nurs* 2013;**69**:1428–37. doi:10.1111/jan.12000

For peer review only

56 Rhodes T, Wagner K, Strathdee SA, *et al.* Structural Violence and Structural Vulnerability Within the Risk Environment: Theoretical and Methodological Perspectives for a Social Epidemiology of HIV Risk Among Injection Drug Users and Sex Workers. In: O'Campo P, Dunn JR, eds. *Rethinking Social Epidemiology: Towards a Science of Change*. Dordrecht: : Springer Netherlands 2012. 205–30. doi:10.1007/978-94-007-2138-8_10

57 Lambdin BH, Bluthenthal RN, Zibbell JE, *et al.* Associations between perceived illicit fentanyl use and infectious disease risks among people who inject drugs. *Int J Drug Policy* 2019;**74**:299–304. doi:10.1016/j.drugpo.2019.10.004

58 Linton SL, Celentano DD, Kirk GD, *et al.* The longitudinal association between homelessness, injection drug use, and injection-related risk behavior among persons with a history of injection drug use in Baltimore, MD. *Drug Alcohol Depend* 2013;**132**:457–65. doi:10.1016/j.drugalcdep.2013.03.009

59 Genberg BL, Gange SJ, Go VF, *et al.* Trajectories of Injection Drug Use Over 20 Years (1988–2008) in Baltimore, Maryland. *Am J Epidemiol* 2011;**173**:829–36. doi:10.1093/aje/kwq441

60 Bundle N, Bubba L, Coelho J, *et al.* Ongoing outbreak of invasive and non-invasive disease due to group A Streptococcus (GAS) type emm66 among homeless and people who inject drugs in England and Wales, January to December 2016. *Euro Surveill* 2017;**22**. doi:10.2807/1560-7917.ES.2017.22.3.30446

61 Parikh MP, Octaria R, Kainer MA. Methicillin-Resistant Staphylococcus aureus Bloodstream Infections and Injection Drug Use, Tennessee, USA, 2015–2017 - Volume 26, Number 3—March 2020 - Emerging Infectious Diseases journal - CDC. *Emerg Infect Dis* 2020;**26**:446–53. doi:10.3201/eid2603.191408

62 Greenhalgh T, Peacock R. Effectiveness and efficiency of search methods in systematic reviews of complex evidence: audit of primary sources. *BMJ* 2005;**331**:1064–5. doi:10.1136/bmj.38636.593461.68

63 Krüsi A, Wood E, Montaner J, *et al.* Social and structural determinants of HAART access and adherence among injection drug users. *Int J Drug Policy* 2010;**21**:4–9. doi:10.1016/j.drugpo.2009.08.003

64 Milloy M-J, Marshall B, Kerr T, *et al.* Social and structural factors associated with HIV disease progression among illicit drug users: A systematic review. *AIDS* 2012;**26**:1049–63. doi:10.1097/QAD.0b013e32835221cc

65 Alvarez E, Joshi S, Lokker C, *et al.* Protocol: Health programmes and services addressing the prevention and management of infectious diseases in persons who inject drugs in Canada: a systematic integrative review protocol. *BMJ Open* 2020;**10**. doi:10.1136/bmjopen-2019-035188

66 Kudrina I, Puzhko S, Filion KB, *et al.* Effectiveness of Interventions for Prevention of Common Infections in People Who Use Opioids: A Protocol for a Systematic Review of Systematic Reviews (Preprint). *ResearchSquare* Published Online First: 2020. doi:10.21203/rs.3.rs-114546/v1

For peer review only

67 Shah M, Wong R, Ball L, *et al.* Risk factors of infective endocarditis in persons who inject drugs. *Harm Reduct J* 2020;**17**:35. doi:10.1186/s12954-020-00378-z

68 Silverman M, Slater J, Jandoc R, *et al.* Hydromorphone and the risk of infective endocarditis among people who inject drugs: a population-based, retrospective cohort study. *The Lancet Infectious Diseases* 2020;**0**. doi:10.1016/S1473-3099(19)30705-4

69 Ciccarone D, Unick GJ, Cohen J, *et al.* Nationwide Increase in Hospitalizations for Heroin-related Soft Tissue Infections: Associations with Structural Market Conditions. *Drug Alcohol Depend* 2016;**163**:126–33. doi:10.1016/j.drugalcdep.2016.04.009

70 Harris M, Scott J, Wright T, *et al.* Injecting-related health harms and overuse of acidifiers among people who inject heroin and crack cocaine in London: a mixed-methods study. *Harm Reduct J* 2019;**16**:60. doi:10.1186/s12954-019-0330-6

71 Yeung A, Weir A, Austin H, *et al.* Assessing the impact of a temporary class drug order on ethylphenidate-related infections among people who inject drugs in Lothian, Scotland: an interrupted time–series analysis. *Addiction* 2017;**112**:1799–807. doi:https://doi.org/10.1111/add.13898

72 Trayner KMA, McAuley A, Palmateer NE, *et al.* Increased risk of HIV and other drug-related harms associated with injecting in public places: national bio-behavioural survey of people who inject drugs. *Int J Drug Policy* 2020;**77**:102663. doi:10.1016/j.drugpo.2020.102663

73 Hong QN, Fàbregues S, Bartlett G, *et al.* The Mixed Methods Appraisal Tool (MMAT) version 2018 for information professionals and researchers. *Education for Information* 2018;**34**:285–91. doi:10.3233/EFI-180221

74 Hong QN, Pluye P. A Conceptual Framework for Critical Appraisal in Systematic Mixed Studies Reviews. *J Mixed Methods Res* 2019;**13**:446–60. doi:10.1177/1558689818770058

75 Hsieh H-F, Shannon SE. Three Approaches to Qualitative Content Analysis. *Qual Health Res* 2005;**15**:1277–88. doi:10.1177/1049732305276687

76 Bonn M, Palayew A, Bartlett S, *et al.* Addressing the Syndemic of HIV, Hepatitis C, Overdose, and COVID-19 Among People Who Use Drugs: The Potential Roles for Decriminalization and Safe Supply. *J Stud Alcohol Drugs* 2020;**81**:556–60. doi:10.15288/jsad.2020.81.556

77 Brothers TD, Bonn M. Patient-centred care in opioid agonist treatment could improve outcomes. *CMAJ* 2019;**191**:E460–1. doi:10.1503/cmaj.190430

78 Bonn M, Touesnard N, Cheng B, *et al.* Barriers and facilitators to accessing and providing a safe supply of drugs during COVID-19 and beyond: a scoping review. *Submitted*

79 Brothers TD, Fraser J, MacAdam E, *et al.* Implementation and evaluation of a novel, unofficial, trainee-organized hospital addiction medicine consultation service. *Subst Abus* 2020;**Published online ahead of print**. doi:10.1080/08897077.2020.1856291

For peer review only

80 Brothers TD, Webster D. Multidisciplinary infective endocarditis care teams should address substance use disorders and harm reduction services. *Open Heart* 2018;**4**. doi:10.1136/openhrt-2017-000699

81 Brothers TD, Fraser J, Webster D. Caring for people who inject drugs when admitted to hospital. *CMAJ: Canadian Medical Association Journal* 2021;**In press.**

82 Tiako MJN, Mszar R, Brooks C, *et al.* Cardiac surgeons' treatment approaches for infective endocarditis based on patients' substance use history. *Semin Thorac Cardiovasc Surg* Published Online First: 3 December 2020. doi:10.1053/j.semtcvs.2020.11.031

83 An KR, Luc JGY, Tam DY, *et al.* Infective endocarditis secondary to injection drug use: A survey of Canadian cardiac surgeons. *Ann Thorac Surg* 2020;**0**. doi:10.1016/j.athoracsur.2020.12.003

84 Harris M, Rhodes T. Venous access and care: harnessing pragmatics in harm reduction for people who inject drugs. *Addiction* 2012;**107**:1090–6.

85 Pantell MS, Adler-Milstein J, Wang MD, *et al.* A call for social informatics. *J Am Med Inform Assoc* 2020;**27**:1798–801. doi:10.1093/jamia/ocaa175

For peer review only

Table 1. A model of the risk environment tabular summary

	**Microenvironmental**	**Macroenvironmental**
Social environment		
Physical environment		
Economic environment		
Policy environment		

Modified from Rhodes, 2002.[34]

For peer review only

Figure captions

Figure 1. Illustrative schematic of a potential risk environment for injecting-related bacterial and fungal infections, as it may structure or create infection risk during the process of drug injection/consumption. Environmental factors, which are external to individuals, interact to influence individual-level factors and health behaviours across stages of a potential pathway: drug acquisition; drug preparation; drug injection; treatment of superficial injection-site bacterial and fungal infections (e.g. in primary care or emergency departments); treatment of severe/invasive bacterial and fungal infections (e.g. in hospital for intravenous antibiotics and/or surgery); and health outcomes after severe/invasive bacterial and fungal infections (e.g. disability, death).

Figure 2. Illustrative schematic of a potential risk environment for injecting-related bacterial and fungal infections, as it may structure or create infection risk during the process of recognition and adequate treatment of superficial injection-site infections. Environmental factors, which are external to individuals, interact to influence individual-level factors and health behaviours across stages of a potential pathway: drug acquisition; drug preparation; drug injection; treatment of superficial injection-site bacterial and fungal infections (e.g. in primary care or emergency departments); treatment of severe/invasive bacterial and fungal infections (e.g. in hospital for intravenous antibiotics and/or surgery); and health outcomes after severe/invasive bacterial and fungal infections (e.g. disability, death).

For peer review only

For peer review only

1

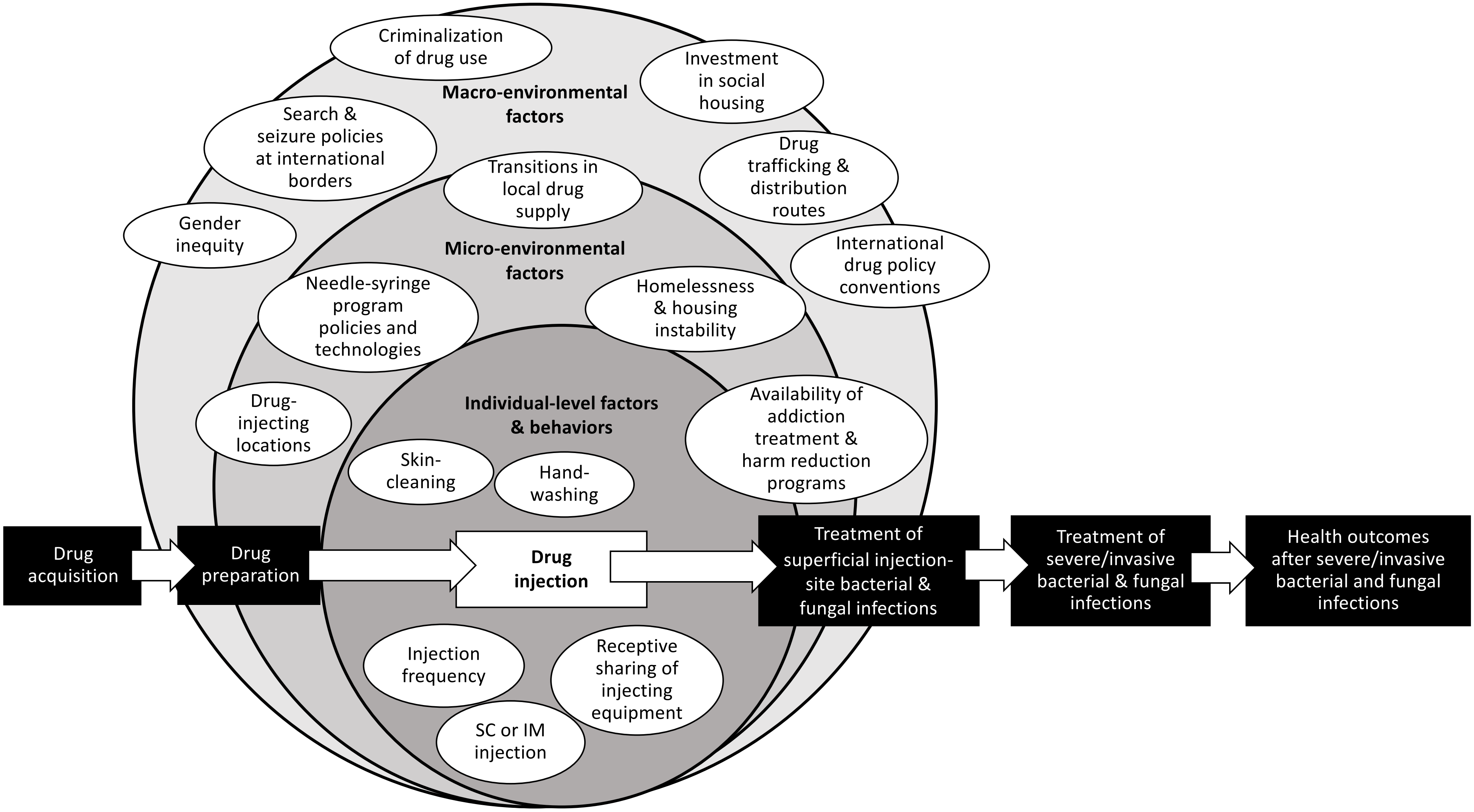
Criminalization of drug use
Investment in social housing
Macro-environmental factors
Search & seizure policies at international borders
Transitions in local drug supply
Drug trafficking & distribution routes
Gender inequity
International drug policy conventions
Micro-environmental factors
Needle-syringe program policies and technologies
Homelessness & housing instability
Individual-level factors & behaviors
Availability of addiction treatment & harm reduction programs
Drug-injecting locations
Skin-cleaning
Hand-washing
Drug acquisition
Drug preparation
Drug injection
Treatment of superficial injection-site bacterial & fungal infections
Treatment of severe/invasive bacterial & fungal infections
Health outcomes after severe/invasive bacterial and fungal infections
Injection frequency
Receptive sharing of injecting equipment
SC or IM injection

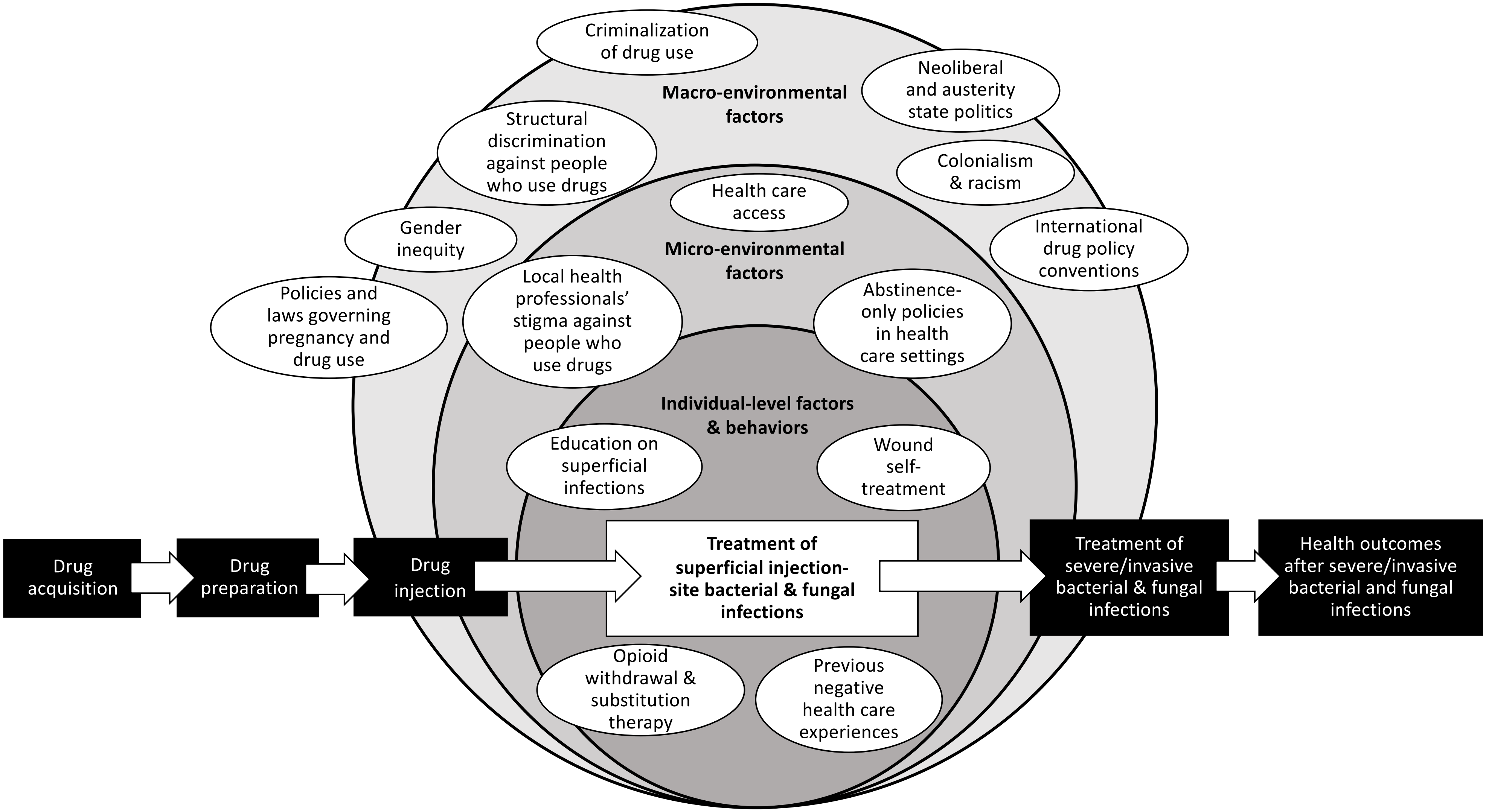
Criminalization of drug use
Neoliberal and austerity state politics
Macro-environmental factors
Structural discrimination against people who use drugs
Colonialism & racism
Health care access
Micro-environmental factors
Gender inequity
International drug policy conventions
Policies and laws governing pregnancy and drug use
Local health professionals' stigma against people who use drugs
Abstinence-only policies in health care settings
Individual-level factors & behaviors
Education on superficial infections
Wound self-treatment
Drug acquisition
Drug preparation
Drug injection
Treatment of superficial injection-site bacterial & fungal infections
Treatment of severe/invasive bacterial & fungal infections
Health outcomes after severe/invasive bacterial and fungal infections
Opioid withdrawal & substitution therapy
Previous negative health care experiences

Supplementary materials for the article:

Social and structural determinants of injecting-related bacterial and fungal infections among people who inject drugs: protocol for a mixed studies systematic review

Thomas D. Brothers[1,2]; Dan Lewer[1]; Matthew Bonn[3]; Duncan Webster[2,4]; Magdalena Harris[5]

[1]UCL Collaborative Centre for Inclusion Health, Institute of Epidemiology & Health Care, University College London (UCL), London, UK
[2]Department of Medicine, Dalhousie University, Halifax, Canada
[3]Canadian Association of People who Use Drugs (CAPUD), Dartmouth, Canada
[4]Division of Infectious Diseases, Saint John Regional Hospital, Saint John, Canada
[5]Department of Public Health, Environments and Society, London School of Hygiene and Topical Medicine (LSHTM), London, UK

This material supplements, but does not replace, the peer-reviewed article in BMJ Open

For peer review only

Supplementary appendix Table 1. Pilot search strategy developed in PubMed on February 1, 2021 (1,752 results)

Concepts	PubMed
People who inject drugs, or drug preparation and injection	("Substance-Related Disorders"[MeSH] OR
	"Substance Abuse, Intravenous"[MeSH] OR
	"Drug Users"[MeSH] OR
	"Needle Sharing"[MeSH] OR
	"people who inject drugs"[tiab] OR "persons who inject drugs"[tiab] OR PWID[tiab] OR
	"people who use drugs"[tiab] OR "persons who use drugs"[tiab] OR PWUD[tiab] OR
	"injection drug"[tiab] OR IDU[tiab] OR
	"intravenous drug"[tiab] OR IVDU[tiab] OR
	"drug abuse"[tiab] OR
	"illicit drugs"[MeSH] OR "illicit drug"[tiab] OR "illegal drug"[tiab] OR
	"Heroin"[MeSH] OR Heroin[tiab] OR
	"Heroin Dependence"[MeSH] OR
	"Opiate use disorder"[tiab] OR "opioid use disorder"[tiab] OR "opiate dependence"[tiab] OR "opioid dependence"[tiab] OR "opiate abuse"[tiab] OR "opioid abuse"[tiab] OR "substance use disorder"[tiab] OR "substance abuse"[tiab] OR
	"Cocaine"[MeSH] OR cocaine[tiab] OR
	"Crack Cocaine"[MeSH] OR
	"groin injecting"[tiab] OR "femoral injecting"[tiab] OR
	"Harm Reduction"[MeSH] OR "harm reduction"[tiab] OR
	"Needle-Exchange Programs"[MeSH] OR

For peer review only

	"needle exchange"[tiab] OR "syringe exchange"[tiab] OR "syringe services"[tiab] OR "syringe program"[tiab] OR
	acidifier*[tiab] OR
	"Opiate Substitution Treatment"[MeSH] OR "opiate substitution"[tiab] OR "opiate agonist"[tiab] OR "opioid substitution"[tiab] OR "opioid agonist"[tiab] OR
	"Medications for opioid use disorder"[tiab] OR MOUD[tiab] OR
	Methadone[tiab] OR
	Buprenorphine[tiab] OR
	Hydromorphone[tiab] OR morphine[tiab] or fentanyl[tiab])
Injecting-related infections	AND
	("injection-related infections"[tiab] OR "injection-related infection"[tiab] OR
	"bacterial infection"[tiab] OR "bacterial infections"[tiab] OR
	Bacteremia[MeSH] OR bacteremia[tiab] OR
	Fungemia[MeSH] OR
	Cellulitis[MeSH] OR cellulitis[tiab] OR
	Abscess[MeSH] OR abscess*[tiab] OR
	"skin infection"[tiab] OR "skin infections"[tiab] OR
	"skin and soft tissue"[tiab] OR SSTI*[tiab] OR
	Endocarditis[MeSH] OR endocarditis[tiab] OR
	Bone Diseases, Infectious[MeSH] OR
	Osteomyelitis[MeSH] OR Osteomyelitis[tiab] OR
	"septic arthritis"[tiab] OR
	Central Nervous System Infections[MeSH] OR

For peer review only

	Gram-Positive Bacterial Infections[MeSH] OR
	Candidiasis[MeSH])
Social and structural determinants, or risk environment	AND
	("risk factor"[tiab] OR "risk factors"[tiab] OR
	correlate*[tiab] OR
	determinant*[tiab] OR
	environment*[tiab] OR
	"social factors"[tiab] or "structural factors"[tiab] OR
	Cohort*[tiab] OR
	Longitudinal[tiab] OR
	Prospective[tiab] OR retrospective[tiab] OR
	Randomized[tiab] OR randomised[tiab] OR
	Comparative[tiab] OR
	Case-control[tiab] OR
	Time-series[tiab] OR
	Survey*[tiab] OR
	Epidemiolog*[tiab] OR
	Qualitative[tiab] OR
	Interview[tiab] OR
	Ethnograph*[tiab] OR
	Mixed-methods[tiab] OR "mixed methods"[tiab] OR
	gender[tiab] OR
	homeless*[tiab] OR

For peer review only

race[tiab] OR racism[tiab] OR

incarcerat*[tiab] OR prison*[tiab] OR criminal*[tiab] OR

stigma*[tiab] OR discrimination[tiab] OR exclusion[tiab])

NOT ("case report"[Title]) NOT ("case series"[Title])

Filter: 2000-Present

For peer review only

Reporting checklist for protocol of a systematic review.

Based on the PRISMA-P guidelines.

Instructions to authors

Complete this checklist by entering the page numbers from your manuscript where readers will find each of the items listed below.

Your article may not currently address all the items on the checklist. Please modify your text to include the missing information. If you are certain that an item does not apply, please write "n/a" and provide a short explanation.

Upload your completed checklist as an extra file when you submit to a journal.

In your methods section, say that you used the PRISMA-Preporting guidelines, and cite them as:

Moher D, Shamseer L, Clarke M, Ghersi D, Liberati A, Petticrew M, Shekelle P, Stewart LA. Preferred Reporting Items for Systematic Review and Meta-Analysis Protocols (PRISMA-P) 2015 statement. Syst Rev. 2015;4(1):1.

		Reporting Item	Page Number
**Title**			
Identification	#1a	Identify the report as a protocol of a systematic review	1
Update	#1b	If the protocol is for an update of a previous systematic review, identify as such	N/A
**Registration**			
	#2	If registered, provide the name of the registry (such as PROSPERO) and registration number	3 & 7
**Authors**			
Contact	#3a	Provide name, institutional affiliation, e-mail address of all protocol authors; provide physical mailing address of corresponding author	1
Contribution	#3b	Describe contributions of protocol authors and identify the guarantor of the review	17
**Amendments**			

For peer review only

	#4	If the protocol represents an amendment of a previously completed or published protocol, identify as such and list changes; otherwise, state plan for documenting important protocol amendments	N/A
**Support**			
Sources	#5a	Indicate sources of financial or other support for the review	17
Sponsor	#5b	Provide name for the review funder and / or sponsor	N/A
Role of sponsor or funder	#5c	Describe roles of funder(s), sponsor(s), and / or institution(s), if any, in developing the protocol	17
**Introduction**			
Rationale	#6	Describe the rationale for the review in the context of what is already known	5-6
Objectives	#7	Provide an explicit statement of the question(s) the review will address with reference to participants, interventions, comparators, and outcomes (PICO)	6
**Methods**			
Eligibility criteria	#8	Specify the study characteristics (such as PICO, study design, setting, time frame) and report characteristics (such as years considered, language, publication status) to be used as criteria for eligibility for the review	8
Information sources	#9	Describe all intended information sources (such as electronic databases, contact with study authors, trial registers or other grey literature sources) with planned dates of coverage	10-11
Search strategy	#10	Present draft of search strategy to be used for at least one electronic database, including planned limits, such that it could be repeated	10-11
Study records - data management	#11a	Describe the mechanism(s) that will be used to manage records and data throughout the review	12
Study records - selection process	#11b	State the process that will be used for selecting studies (such as two independent reviewers) through each phase of the review (that is, screening, eligibility and inclusion in meta-analysis)	12
Study records - data collection process	#11c	Describe planned method of extracting data from reports (such as piloting forms, done independently, in duplicate), any processes for	12

For peer review only

		obtaining and confirming data from investigators	
Data items	#12	List and define all variables for which data will be sought (such as PICO items, funding sources), any pre-planned data assumptions and simplifications	12
Outcomes and prioritization	#13	List and define all outcomes for which data will be sought, including prioritization of main and additional outcomes, with rationale	12
Risk of bias in individual studies	#14	Describe anticipated methods for assessing risk of bias of individual studies, including whether this will be done at the outcome or study level, or both; state how this information will be used in data synthesis	13
Data synthesis	#15a	Describe criteria under which study data will be quantitatively synthesised	13
Data synthesis	#15b	If data are appropriate for quantitative synthesis, describe planned summary measures, methods of handling data and methods of combining data from studies, including any planned exploration of consistency (such as I2, Kendall's τ)	N/A
Data synthesis	#15c	Describe any proposed additional analyses (such as sensitivity or subgroup analyses, meta-regression)	N/A
Data synthesis	#15d	If quantitative synthesis is not appropriate, describe the type of summary planned	13
Meta-bias(es)	#16	Specify any planned assessment of meta-bias(es) (such as publication bias across studies, selective reporting within studies)	N/A
Confidence in cumulative evidence	#17	Describe how the strength of the body of evidence will be assessed (such as GRADE)	13

The PRISMA-P checklist is distributed under the terms of the Creative Commons Attribution License CC-BY 4.0. This checklist was completed on 27. January 2021 using https://www.goodreports.org/, a tool made by the EQUATOR Network in collaboration with Penelope.ai

For peer review only

BMJ Open

Social and structural determinants of injecting-related bacterial and fungal infections among people who inject drugs: protocol for a mixed studies systematic review

Journal:	*BMJ Open*
Manuscript ID	bmjopen-2021-049924.R1
Article Type:	Protocol
Date Submitted by the Author:	28-Jun-2021
Complete List of Authors:	Brothers, Thomas; UCL Institute of Epidemiology and Health Care, UCL Collaborative Centre for Inclusion Health; Dalhousie University, Department of Medicine<br>Lewer, Dan; UCL Institute of Epidemiology and Health Care, UCL Collaborative Centre for Inclusion Health<br>Bonn, Matthew; Canadian Association of People Who Use Drugs, Dartmouth, Nova Scotia,<br>Webster, Duncan; Dalhousie University, Department of Medicine; Saint John Regional Hospital, Division of Infectious Diseases<br>Harris, Magdalena; London School of Hygiene & Tropical Medicine, Department of Public Health, Environments and Society
**Primary Subject Heading**:	Addiction
Secondary Subject Heading:	Public health, Epidemiology, Health services research, Infectious diseases, Sociology
Keywords:	Substance misuse < PSYCHIATRY, INFECTIOUS DISEASES, SOCIAL MEDICINE, Public health < INFECTIOUS DISEASES, PUBLIC HEALTH, EPIDEMIOLOGY

SCHOLARONE™
Manuscripts

I, the Submitting Author has the right to grant and does grant on behalf of all authors of the Work (as defined in the below author licence), an exclusive licence and/or a non-exclusive licence for contributions from authors who are: i) UK Crown employees; ii) where BMJ has agreed a CC-BY licence shall apply, and/or iii) in accordance with the terms applicable for US Federal Government officers or employees acting as part of their official duties; on a worldwide, perpetual, irrevocable, royalty-free basis to BMJ Publishing Group Ltd ("BMJ") its licensees and where the relevant Journal is co-owned by BMJ to the co-owners of the Journal, to publish the Work in this journal and any other BMJ products and to exploit all rights, as set out in our licence.

The Submitting Author accepts and understands that any supply made under these terms is made by BMJ to the Submitting Author unless you are acting as an employee on behalf of your employer or a postgraduate student of an affiliated institution which is paying any applicable article publishing charge ("APC") for Open Access articles. Where the Submitting Author wishes to make the Work available on an Open Access basis (and intends to pay the relevant APC), the terms of reuse of such Open Access shall be governed by a Creative Commons licence – details of these licences and which Creative Commons licence will apply to this Work are set out in our licence referred to above.

Other than as permitted in any relevant BMJ Author's Self Archiving Policies, I confirm this Work has not been accepted for publication elsewhere, is not being considered for publication elsewhere and does not duplicate material already published. I confirm all authors consent to publication of this Work and authorise the granting of this licence.

For peer review only

Social and structural determinants of injecting-related bacterial and fungal infections among people who inject drugs: protocol for a mixed studies systematic review

Thomas D. Brothers[1,2]; Dan Lewer[1]; Matthew Bonn[3]; Duncan Webster[2,4]; Magdalena Harris[5]

[1]UCL Collaborative Centre for Inclusion Health, Institute of Epidemiology & Health Care, University College London (UCL), London, UK
[2]Department of Medicine, Dalhousie University, Halifax, Canada
[3]Canadian Association of People who Use Drugs (CAPUD), Dartmouth, Canada
[4]Division of Infectious Diseases, Saint John Regional Hospital, Saint John, Canada
[5]Department of Public Health, Environments and Society, London School of Hygiene and Topical Medicine (LSHTM), London, UK

Address correspondence to:
Thomas D. Brothers, MD
483 Bethune Building
1276 South Park St.
Halifax, NS B3H 2Y9
thomas.brothers@dal.ca

Version #2

Word count: 3409

Email & ORCiD:
Thomas D. Brothers: thomas.brothers@dal.ca; 0000-0002-5570-5556
Dan Lewer: d.lewer@ucl.ac.uk; 0000-0003-3698-7196
Matthew Bonn: matthewbonn00@gmail.com; 0000-0002-6406-0171
Duncan Webster: duncan.webster@horizonnb.ca; 0000-0001-7692-7150
Magdalena Harris: magdalena.harris@lshtm.ac.uk; 0000-0001-8718-8226

For peer review only

ABSTRACT

Introduction: Injecting-related bacterial and fungal infections are a common complication among people who inject drugs (PWID), associated with significant morbidity and mortality. Invasive infections, including infective endocarditis, appear to be increasing in incidence. To date, preventive efforts have focused on modifying individual-level risk behaviours (e.g. handwashing, skin cleaning) without much success in reducing the population-level impact of these infections. Learning from successes in HIV prevention, there may be great value in looking beyond individual-level risk behaviours to the social determinants of health. Specifically, the risk environment conceptual framework identifies how social, physical, economic, and political environmental factors facilitate and constrain individual behaviour, and therefore influence health outcomes. Understanding the social and structural determinants of injecting-related bacterial and fungal infections could help to identify new targets for prevention efforts in the face of increasing incidence of severe disease.

Methods and analysis: This is a protocol for a systematic review. We will review studies of PWID and investigate associations between risk factors (both individual-level and social/structural-level) and the incidence of hospitalization or death due to injecting-related bacterial infections (skin and soft-tissue infections, bacteremia, infective endocarditis, osteomyelitis, septic arthritis, epidural abscess, and others). We will include quantitative, qualitative, and mixed methods studies. Using directed content analysis, we will code risk factors for these infection-related outcomes according to their contributions to the risk environment in type (social, physical, economic, or political) and level (micro- or macro-environmental). We will also code and present risk factors at each stage in the process of drug acquisition, preparation, injection, superficial infection care, severe infection care or hospitalization, and outcomes after infection or hospital discharge.

For peer review only - http://bmjopen.bmj.com/site/about/guidelines.xhtml

Ethics and dissemination: As an analysis of published literature, no ethics approval is required. The findings will inform a research agenda to develop and implement social/structural interventions aimed at reducing the burden of disease.

PROSPERO registration number: CRD42021231411

Keywords: injection drug use; injecting drug use; cellulitis; abscess; endocarditis; social determinants; risk environment

For peer review only

Strengths and limitations of this study

- Injecting-related bacterial and fungal infections are a common problem among people who inject drugs (PWID), associated with significant morbidity and mortality.
- Understanding the social and structural determinants of injecting-related bacterial and fungal infections could help to identify new targets for prevention efforts, in the face of increasing incidence of severe disease.
- People with lived and living expertise of injection drug use, as well as practicing clinicians caring for PWID with injecting-related infections and experienced qualitative, quantitative, and mixed methods researchers, were involved in the development of the protocol and will participate in analysis and synthesis.
- Reviewing quantitative, qualitative, and mixed methods studies enables a broad scope and the inclusion of important contextual information that may not be captured in quantitative studies alone.
- This review only includes studies with infection-related outcomes, and so may not include some studies with risk factors for infection (e.g. hand-washing, skin-cleaning) as outcomes.

For peer review only

For peer review only - http://bmjopen.bmj.com/site/about/guidelines.xhtml

INTRODUCTION

Injecting-related bacterial infections are very common among people who inject drugs (PWID), with up to 39% of PWID reporting skin infections within the past month and 70% reported lifetime prevalence.[1] If not effectively treated, superficial infections can progress and spread through the bloodstream, leading to life-threatening infections such as infective endocarditis, osteomyelitis, septic arthritis, or epidural abscess.[2] Pathogenic bacteria or fungi may be introduced directly into the bloodstream as endogenous skin flora, or from non-sterile injecting equipment or contaminated drugs.[3] As a result, PWID face extremely high risk of disability or death from severe bacterial and fungal infections that require hospitalization for antibiotics and/or surgery.[4–9] The incidence of severe bacterial and fungal infections is increasing among PWID in Canada[10–12], the United Kingdom [13], the USA [14–19], Australia [20], and Sweden.[21] A recent modelling study suggests that one in five PWID in the USA may die of infective endocarditis alone over the next 10 years.[19] Better approaches to prevention are urgently needed.

To date, prevention efforts for injecting-related bacterial and fungal infections have primarily focused on changing individual-level risk behaviours,[1,22] including hand-washing before drug preparation,[23] skin-cleaning before injecting,[24] and avoiding subcutaneous or intramuscular injections.[25] While individual-level interventions may be effective for people who due to their socioeconomic situation can adopt these practices,[26,27] they have not translated into reductions in the population-level incidence of severe bacterial and fungal infections, which continue to rise.

An appreciation for the importance of social and structural determinants of health has emerged from the understanding that individual health behaviours are shaped by contextual factors.[28–32] The "risk environment" conceptual framework, as developed by Rhodes and others,[33–36] describes how

For peer review only - http://bmjopen.bmj.com/site/about/guidelines.xhtml

interactions between social and structural factors external to the individual influence individual behaviours, and therefore structure or create health harms.[37] The risk environment framework has informed clinical and public health efforts at reducing other drug-related harms, including human immunodeficiency virus (HIV) transmission,[33,36,38] hepatitis C virus (HCV) treatment,[39] and drug overdoses.[29,40] Better understanding of the social determinants of health can help to shift beliefs about responsibility and risk from individual behaviours to the places and social situations in which individuals exist, and inform the development of innovative interventions addressing both social and individual-level factors.[36,41]

Like other drug-related harms, risk for injecting-related bacterial and fungal infections also likely reflects contributions of multiple, interacting factors external to individuals that influence risk behaviours and therefore shape health outcomes. For example, homelessness may constrain an individual's ability to wash their hands or use sterile water for injecting,[42] and policy constraints on needle and syringe programs (NSP) (from criminalisation to reduced operating hours) create a situation in which an individual is more likely to reuse a blunted or contaminated needle. Stigma and criminalization of people who use drugs may keep people away from primary health care, causing superficial bacterial infections to remain untreated and progress to enter the bloodstream. In response to the alarming increases in incidence, calls to enhance understanding of the social determinants of injecting-related bacterial and fungal infections have emerged from both PWID[43] and academic communities.[2,4,26,44] Learning from the successes of HIV prevention efforts in particular, mapping the risk environment for injecting-related bacterial and fungal infections could inform new prevention efforts that target social and structural causes.[45,46]

Objectives

For peer review only

For peer review only - http://bmjopen.bmj.com/site/about/guidelines.xhtml

In order to better understand risk for injecting-related bacterial and fungal infections among PWID, we seek to conduct a systematic mixed studies review (of quantitative, qualitative, and mixed methods evidence) to identify social and structural determinants of these infections among PWID, informed by the risk environment framework. The proposed review will seek to answer the question, “Among people who inject drugs (PWID), what social and structural factors are associated with the development, treatment, and outcomes of injecting-related bacterial and fungal infections?”

METHODS AND ANALYSIS

This systematic review protocol follows the Preferred Reporting Items for Systematic Review and Meta-Analysis Protocols (PRISMA-P) guideline[47,48] and is informed by guidance on conducting systematic reviews of association (etiology) by the Joanna Briggs Institute[49] and on conducting and reporting mixed studies reviews by Pluye and colleagues[50,51] and by Tricco and colleagues.[52] We plan to conduct this study over 12 months following the initial full database search on February 18, 2021.

“Mixed studies reviews” incorporate synthesis of quantitative, qualitative, and mixed methods studies; they are one of several emerging methods for systematically incorporating evidence from diverse methodologies (e.g. integrative review, realist review, meta-narrative review, critical interpretive synthesis).[50,52,53] Mixed studies reviews use a mixed methods approach.[54,55] They are particularly useful for research questions like ours that require detailed contextual information available from qualitative and mixed methods studies, and for synthesis plans like ours that incorporate a qualitative content analysis directed by existing theories and conceptual frameworks.[50,52]

For peer review only

This systematic mixed studies review protocol is registered with PROSPERO (registration number CRD42021231411). We have searched the PROSPERO registry of systematic reviews and found none in progress related to our objectives.

Conceptual framework

The risk environment framework is the most prominent social-ecological model for substance use research; it comprises risk factors external to individuals, considering types and levels of environmental influence.[34,35,37,56] As first developed in the context of HIV prevention, the risk environment framework describes four different types of environmental influences: social, physical, economic, and policy. These can occur at two different levels, microenvironmental or macroenvironmental.[38] Microenvironmental factors operate at the level of interpersonal relationships, community and group norms, and institutional or organizational responses.[36] This could include local norms about the culture of substance use and acceptability of receptive sharing or reuse of potentially contaminated injecting equipment among PWID (a social factor), or increasing housing prices (an economic factor) contributing to homelessness and lack of access to soap and water (physical factors). Macroenvironmental factors operate at the level of states, societies, and laws, and interact with microenvironmental factors.[36] This could include state policing crackdowns on heroin importation leading instead to increased importation of fentanyl (a policy factor), which has a shorter half-life and associated risks of increased injecting frequency.[57]

Eligibility criteria

Informed by the Population, Exposures, Outcomes (PEO) approach,[49] we will include peer-reviewed papers describing eligible studies according to the following criteria.

For peer review only

Study designs

We will include studies measuring quantitative associations between exposures and outcomes of interest (as described below), and studies reporting qualitative data on relationships between experiences of exposures and outcomes. We will exclude case-reports and case series that do not include analyses of association, and we will exclude reviews, commentaries, and editorials, as they do not include original data.

Participants

We will include studies examining PWID of any age or nationality. By PWID, we are referring to people who inject opioids (e.g. heroin, fentanyl, hydromorphone, morphine), stimulants (e.g. cocaine, methamphetamines), or other psychoactive substances via intravenous, intramuscular, or subcutaneous routes. We expect there to be differences between studies in how PWID are operationally defined (e.g. ever injected, recently injected in past year or past 3 months, etc.).[1] Taking a broad perspective, we will include studies that identify participants as PWID at risk for injecting-related infections, and not limit this to self-reported current or recent injecting. Many PWID transition back and forth between injection and non-injection routes of consumption,[58,59] and PWID who have stopped injecting may experience persistently increased risk for invasive infections such as endocarditis, due to prior damage to vasculature and heart valves.[2] Studies that focus only on people who inject performance-enhancing drugs (e.g. anabolic steroids) or gender-affirming hormones will be excluded as these are not psychoactive substances.

Exposures

We will include studies addressing risk factors or determinants for injecting-related bacterial and fungal infections among PWID, taking a broad perspective. In addition to studies that directly survey individual

For peer review only

participants about potential risk factors, we will consider studies investigating potential associations with social or structural risk factors at micro-environmental and macro-environmental levels. We will also include studies of interventions that aim to reduce risk, including educational, behavioural, or structural (e.g. community mobilization or policy change) interventions.[26,27,46] Studies that only report on individual-level risk factors (e.g. skin-cleaning) will be included in the search, and then classified separately in the data synthesis.

Outcomes

Outcomes of interest include incidence of, hospitalization with, or mortality due to, injecting-related bacterial and fungal infections. Bacterial and fungal infections include skin and soft-tissue infections (cellulitis, abscess, necrotizing fasciitis), bloodstream infections (bacteremia), vascular infections (endocarditis, septic or suppurative phlebitis), bone and joint infections (osteomyelitis, septic arthritis, discitis), and central nervous system infections (epidural abscess, brain abscess, meningitis, encephalitis). We will also include as outcomes, diseases caused by pathogenic bacteria and fungi associated with drug supply, preparation, and injecting practices, including tetanus,[22] botulism[3], invasive group A streptococcal disease,[60] as well as methicillin-sensitive *Staphylococcus aureus* (MSSA) and methicillin-resistant *Staphylococcus aureus* (MRSA).[61] We will exclude studies identifying only non-injecting related bacterial and fungal infections as outcomes, such as tuberculosis, pneumonia, chlamydia, or gonorrhea, and bacterial infections that are primarily sexually transmitted, e.g. syphilis.

We will extract all outcomes as reported. We expect there to be many different methods for identifying these outcomes, including participant self-report of infection or hospitalization, administrative records of hospital admission, and mortality records from vital statistics.

For peer review only

For peer review only - http://bmjopen.bmj.com/site/about/guidelines.xhtml

Time frame, setting, and language

We will include studies published between January 1, 2000, and the planned search date, February 18, 2021, to capture contemporary research that would be more likely to inform policy and clinical practice. There will be no restrictions by study setting. We will include articles in English and French. A list of potentially relevant titles reported in other languages will be provided as an appendix.

Information sources

We will search PubMed, EMBASE, Scopus, CINAHL, and PsycINFO databases. Electronic database searches will be supplemented by manually reviewing reference lists of included studies, and forward "snowball" searching by identifying articles that cite included studies.[62] We will also circulate a bibliography of the included articles to the systematic review team, which includes people with lived (past) and living (current) expertise of injection drug use and clinicians who care for people with injection-associated infections. We will include articles identified from the personal files of the systematic review team that were not identified in the bibliography.

Search strategy

Our search strategy is informed by several key existing systematic reviews or review protocols. A 2017 systematic review identified prevalence and individual-level risk factors for injecting-related injuries and diseases among PWID, including bacterial infections.[1] Previous systematic reviews have applied the risk environment framework to understand HIV transmission[33], HIV[63] and HCV[39] treatment access, HIV disease progression,[64] providing or receiving assistance with the transition to injection drug use,[65] and "safer environment interventions"[41] among PWID. Two published protocols of systematic reviews in progress focused on health services and interventions to treat or prevent viral and bacterial infections among PWID.[66,67]

For peer review only - http://bmjopen.bmj.com/site/about/guidelines.xhtml

The final search strategy will be developed by our review team in consultation with health information specialist-librarians with systematic review experience. We conducted a pilot search in PubMed and validated by checking for inclusion of key, recent studies known to the authors that assessed either risk factors for severe bacterial or fungal infections among PWID,[68,69] social/structural determinants of bacterial infections among PWID,[42,70–73] or complex interventions to reduce risk of bacterial infections among PWID[26] (Appendix Table 1). This validation process led to several iterative changes, including specific search terms related to "acidifiers" and "groin injecting". See Table 1 for the draft search strategy in PubMed, which identified 1,752 potentially relevant sources on February 1, 2021.

Data management and reference selection

Search results will be uploaded into Covidence, a cloud-based software program, where they can be automatically checked for de-duplication. Two reviewers will screen titles and abstracts against the inclusion criteria. We will obtain full text reports for all titles that appear to meet the inclusion criteria, or for where there is uncertainty. Two reviewers will screen these full text reports and mark each as included or excluded, recording reasons for exclusion. We will resolve discrepancies through discussion.

Data collection process

The team will develop and pilot-test a data extraction form, which can be applied in Covidence. We will extract information on:

- Study date (or publication date, if study date unavailable)
- Study country/region
- Methodology (quantitative, qualitative, or mixed methods)
- Study design

For peer review only

- Sample size and demographic characteristics (age, gender, race/ethnicity)
- Inclusion criteria and definition of PWID
- Sampling methods and recruitment setting (e.g. community recruitment, needle exchange clients, clinic, hospital, etc.)
- Outcomes reported (incident infection/diagnosis, clinic visit, emergency department visit, hospital admission, or death)
- Outcome operational criteria (self-reported, medical record review, administrative data)
- Infections reported (skin and soft-tissue infections at injection sites [e.g. abscess, cellulitis], bacteremia, infective endocarditis, osteomyelitis, septic arthritis, or other)[1]
- Exposures, risk factors, or interventions assessed (including operational criteria)
- Measures of association between risk factors and outcomes (for quantitative and mixed methods studies)
- Summary of findings
- Implications for policy and/or practice, as reported by authors[66]
- Implications for research, as reported
- Gaps identified, as reported

Critical appraisal/Risk of bias assessment

We will apply a formal, validated critical appraisal tool for mixed studies reviews, the Mixed Methods Appraisal Tool (MMAT), 2018 edition, which is designed for use with quantitative, qualitative, and mixed methods studies.[74,74,75] It includes five core quality criteria for five categories of study designs: (a) qualitative, (b) randomized controlled/interventional, (c) nonrandomized controlled, (d) quantitative descriptive, and (e) mixed methods. This tool does not assign an overall quality score; rather, we will consider each item in the risk of bias assessment independently. Due to the broad scope of this review,

For peer review only

we will not exclude studies deemed low quality. Study quality and risk of bias will inform interpretation of the data.

Data synthesis and analysis

We will perform data synthesis and analysis over several stages. We will perform a quantitative tabular synthesis of included studies, by year; country/region; methodology (quantitative, qualitative, or mixed methods), infection type (cellulitis, endocarditis, etc.); and outcome (incident infection, care of superficial infection, care of severe infection/hospitalization, or outcomes after infection).

We will then conduct a qualitative, directed content analysis, as described by Hsieh and Shannon,[76] to map the risk environment for injecting-related bacterial and fungal infections. We will first code each included exposure or risk factor according to the risk environment framework as individual-level or social/structural. Social/structural factors are those that are external to the individual, and together make up the risk environment. We will then code each social/structural factor according to the type (social, economic, political, or physical) and level (macro-environmental or micro-environmental) of the risk environment. We will use a hybrid deductive-inductive approach, as described by Pluye and Hong;[50] we will start with the existing risk environment conceptual framework but allow for new concepts related to the classification of specific factors to emerge. To improve reliability, coding will be performed in tandem by a reviewer pair for three studies, with discrepancies resolved through discussion. Coding will then be discussed with the whole review team to identify improvements to the coding approach and framework. The remaining studies will be coded by one reviewer. We will present a tabular synthesis categorizing each social/structural factor according to type and level in the risk environment framework,[49] and a narrative synthesis of the existing evidence for each social/structural factor.[39] See Table 1 for the model table framework.

For peer review only - http://bmjopen.bmj.com/site/about/guidelines.xhtml

In order to explore how different macro- and micro-environmental factors influence individual-level risk behaviours, we will present a narrative synthesis of identified individual-level and social/structural factors affecting each step of a potential pathway from (a) drug acquisition; (b) drug preparation; (c) drug injection; (d) development of and care for superficial infections (e.g. self-treatment or primary/emergency department care); (e) development of and care for severe or invasive infections (e.g. self-care or hospital care); and (f) outcomes after infection or hospital discharge, including infection-related mortality.[2] See Figure 1 and Figure 2 for illustrative schematics of how macro- and micro-environmental factors influence individual-level factors and may increase risks of injecting-related bacterial infections at two different stages in the pathway from drug acquisition to outcomes after severe/invasive infections. We plan to develop and refine these figures to reflect findings of the review. Note that because this review includes only studies with infection-related outcomes, we may not identify or include all published studies that identify determinants of intermediate risk factors or behaviours; for example some studies identifying determinants of skin cleaning before injecting may not be included in this review if they do not consider infection-related outcomes.

If we identify sufficient quantitative data, we will conduct a meta-analysis of summary effects of association between social/structural factors (e.g. homelessness) and injecting-related infection outcomes among PWID (e.g. hospitalization with any severe injecting-related bacterial or fungal infection) identified in our content analysis. Where available, we will extract measures of association adjusted for confounding, including point estimates and standard errors. To assess feasibility, we will evaluate clinical and methodological heterogeneity by comparing study design, sample characteristics, and operational definitions of exposures and outcome [65,77]. We will measure statistical variability between studies using I^2 statistics.[65,77,78]

For peer review only - http://bmjopen.bmj.com/site/about/guidelines.xhtml

Patient and public involvement

The research team includes people with lived/living expertise of injection drug use and clinicians caring for PWID with bacterial and fungal injections. The research questions and analysis approach have been designed collaboratively, and build on existing relationships between authors with lived/living expertise and academic/medical expertise.[79–81] The topic was inspired by the deaths of friends and patients to injecting-related bacterial infections, and a recognition of the need to look broader than individual-level interventions.[82–84]

Ethics and dissemination

As this is a secondary analysis of published literature, no ethics approval is required.

By mapping the risk environment for injecting-related bacterial and fungal infections among PWID, this study aims to identify opportunities for new policy and practice approaches to prevention. This may include highlighting the importance of scaling up access to existing interventions (e.g. needle/syringe distribution; opioid agonist treatment) or identifying opportunities for novel combined social and clinical interventions (e.g. providing Housing First along with antibiotics and addiction treatment for PWID hospitalized with invasive infections). Our evidence synthesis may identify opportunities for "structural interventions", which promote health by altering the social-structural context which influence health.[46,85] Examples include the potential impacts of policy or law changes (e.g. decriminalizing substance use may combat stigma and facilitate access to primary care of injecting-related infections) or of community mobilization (e.g. organized PWID empowered to effectively demand safe and welcoming hospital care).[44,86,87]

For peer review only

For peer review only - http://bmjopen.bmj.com/site/about/guidelines.xhtml

Incidence of HIV and HCV infections are increasing in parallel with injecting-related bacterial and fungal infections among PWID, in the context of the North American overdose crisis. While our review focuses only on injecting-related bacterial and fungal infections, we expect to find that these outcomes share many overlapping social-structural correlates.[29,45,79,85,88] Secular increases in drug-related infections and overdoses over the past decade have been describes as a “syndemic”, with shared, fundamental causes rooted in the “War on Drugs” and austerity politics.[29,42,79,85] Accordingly, findings from our study may be able to inform risk reduction interventions for multiple health outcomes among PWID. The COVID-19 pandemic has accelerated the overdose death crisis, deepened poverty and marginalization, and interrupted access to harm reduction services[79,85] – the consequential impacts on injecting-related bacterial and fungal infections, and potential for specific interventions, is not yet clear.

The risk environment approach may also help to shift beliefs regarding responsibility and risk from individual behaviours to social and structural causes, which may change attitudes of health care professionals and systems regarding treatment of severe infections, including procedures such as heart valve replacement surgeries for injecting-related infective endocarditis.[89–91] This may also inform educational and behaviour-change interventions, highlighting the importance of addressing more immediate, pragmatic priorities (e.g. housing and income, or vein access and care) that may also have benefits in infection risk reduction.[92] Extending further concepts from the HIV prevention social science literature, we are also interested in how our findings may relate to interventions in social networks.[93] Social relationships within networks of PWID influence the likelihood of individual risk or protective behaviours (e.g. needle re-use; skin cleaning). While bacterial and fungal infections are generally not transmitted through blood, as is HIV or HCV, PWID in the same network may experience

For peer review only - http://bmjopen.bmj.com/site/about/guidelines.xhtml

transmission of colonization with pathogenic bacteria (e.g. MRSA)[94] or heightened risk of accessing a shared, contaminated drug supply (e.g. outbreaks of botulism[3,95] or anthrax[96]).

By emphasizing the potential importance of social and structural factors, the findings will also inform future epidemiological research using large-scale linked administrative, data linking individual and clinical risk factors with information on social factors.[69,97] Knowledge translation activities will include peer-reviewed academic publications and conference presentations, meetings with drug user groups/unions and harm reduction programs, and also communications to the lay public in targeted outlets.

For peer review only

For peer review only - http://bmjopen.bmj.com/site/about/guidelines.xhtml

Authors contributions

This study was conceived by TDB, DL, MB, DW, and MH. The pilot search strategy was developed by TDB, and revised with input from DL and MD. TDB, DL, and MB will be involved in the data collection for this study. All authors will be involved in data interpretation and analysis. TDB wrote the first draft of this manuscript. All authors provided critical feedback and intellectual input. All authors provided their final approval for the publication of this version of the manuscript and agree to be accountable for all aspects of this work.

Funding statement: TDB is supported by the Dalhousie University Internal Medicine Research Foundation Fellowship, Killam Postgraduate Scholarship, Ross Stewart Smith Memorial Fellowship in Medical Research, and Clinician Investigator Program Graduate Stipend (all from Dalhousie University Faculty of Medicine), a Canadian Institutes of Health Research Fellowship [CIHR-FRN# 171259], and through the Research in Addiction Medicine Scholars (RAMS) Program (National Institutes of Health/National Institute on Drug Abuse; R25DA033211). DL is funded by a National Institute of Health Research Doctoral Research Fellowship [DRF-2018-11-ST2-016]. MB was supported in this work via the Ross Stewart Smith Memorial Fellowship in Medical Research, from Dalhousie University Faculty of Medicine. MH is funded by a National Institute of Health Research Career Development Fellowship [CDF-2016-09-014]. The views expressed are those of the author(s) and not necessarily those of the NHS, the NIHR or the Department of Health and Social Care. These funders had no role in the conduct or reporting of the research.

Declaration of interests

For peer review only

For peer review only - http://bmjopen.bmj.com/site/about/guidelines.xhtml

MB reports personal fees from AbbVie, a pharmaceutical research and development company, and grants and personal fees from Gilead Sciences, a research-based biopharmaceutical company, outside of the submitted work. The other authors have no conflicts to declare.

Acknowledgements

We appreciate the contributions to science of all participants in the included studies. TDB, MB, and DW live and work in Mi'kma'ki and along the Wolastoq, the ancestral and unceded territory of the Mi'kmaq and the Wolastoqiyik. We are all Treaty people.

We thank Prof. Andrew Hayward for helpful suggestions on the synthesis and analysis plan. We thank the Reviewers and Editor for helpful suggestions that improved the manuscript.

For peer review only

For peer review only - http://bmjopen.bmj.com/site/about/guidelines.xhtml

REFERENCES

1 Larney S, Peacock A, Mathers BM, *et al.* A systematic review of injecting-related injury and disease among people who inject drugs. *Drug Alcohol Depend* 2017;**171**:39–49. doi:10.1016/j.drugalcdep.2016.11.029

2 Robertson R, Broers B, Harris M. Injecting drug use, the skin and vasculature. *Addiction* 2021;**116(7)**:1914-1924. doi:10.1111/add.15283

3 Trayner KMA, Weir A, McAuley A, *et al.* A pragmatic harm reduction approach to manage a large outbreak of wound botulism in people who inject drugs, Scotland 2015. *Harm Reduct J* 2018;**15**:36. doi:10.1186/s12954-018-0243-9

4 Wurcel AG. Drug-Associated Infective Endocarditis Trends: What's All the Buzz About? *Ann Intern Med* 2019;**170**:68. doi:10.7326/M18-3026

5 Oh DHW, Wurcel AG, Tybor DJ, *et al.* Increased Mortality and Reoperation Rates After Treatment for Septic Arthritis of the Knee in People Who Inject Drugs: Nationwide Inpatient Sample, 2000-2013. *Clin Orthop* 2018;**476**:1557–65. doi:10.1097/01.blo.0000534682.68856.d8

6 Serota DP, Barocas JA, Springer SA. Infectious complications of addiction: A call for a new subspecialty within infectious diseases. *Clin Infect Dis* 2020;**70**:968–72. doi:10.1093/cid/ciz804

7 Lewer D, Freer J, King E, *et al.* Frequency of healthcare utilisation by adults who use illicit drugs: a systematic review and meta-analysis. *Addiction* 2020;**115**:1011–23. doi:10.1111/add.14892

8 Lewer Dan, Hope VD, Harris Magdalena, *et al.* Incidence and treatment costs of severe bacterial infections among people who inject heroin: A cohort study in South London, England. *Drug Alcohol Depend* 2020;**212**:108057. doi:10.1016/j.drugalcdep.2020.108057

9 Wright T, Hope V, Ciccarone D, *et al.* Prevalence and severity of abscesses and cellulitis, and their associations with other health outcomes, in a community-based study of people who inject drugs in London, UK. *PLOS ONE* 2020;**15**:e0235350. doi:10.1371/journal.pone.0235350

10 Weir MA, Slater J, Jandoc R, *et al.* The risk of infective endocarditis among people who inject drugs: a retrospective, population-based time series analysis. *CMAJ* 2019;**191**:E93–9. doi:10.1503/cmaj.180694

11 Coverdale N, Brogly S, Payne D, *et al.* Rates of Infective Endocarditis in Substance Use Disorder and Associated Costs in Ontario. *Can J Addict* 2019;**10**:36–43. doi:10.1097/CXA.0000000000000053

12 Mosseler K, Materniak S, Brothers TD, *et al.* Epidemiology, microbiology, and clinical outcomes among patients with intravenous drug use-associated infective endocarditis in New Brunswick. *Can J Cardiol Open* 2020;**2**:379–85. doi:https://doi.org/10.1016/j.cjco.2020.05.002

For peer review only

For peer review only - http://bmjopen.bmj.com/site/about/guidelines.xhtml

13 Lewer D, Harris M, Hope V. Opiate Injection–Associated Skin, Soft Tissue, and Vascular Infections, England, UK, 1997–2016. *Emerg Infect Dis* 2019;**23**:1400–3. doi:10.3201/eid2308.170439

14 Bates MC, Annie F, Jha A, *et al.* Increasing incidence of IV-drug use associated endocarditis in southern West Virginia and potential economic impact. *Clin Cardiol* 2019;**42**:432–7. doi:10.1002/clc.23162

15 Deo SV, Raza S, Kalra A, *et al.* Admissions for Infective Endocarditis in Intravenous Drug Users. *J Am Coll Cardiol* 2018;**71**:1596–7. doi:10.1016/j.jacc.2018.02.011

16 Fleischauer AT. Hospitalizations for Endocarditis and Associated Health Care Costs Among Persons with Diagnosed Drug Dependence — North Carolina, 2010–2015. *MMWR Morb Mortal Wkly Rep* 2017;**66**. doi:10.15585/mmwr.mm6622a1

17 Schranz AJ, Fleischauer A, Chu VH, *et al.* Trends in Drug Use–Associated Infective Endocarditis and Heart Valve Surgery, 2007 to 2017: A Study of Statewide Discharge Data. *Ann Intern Med* 2019;**170**:31. doi:10.7326/M18-2124

18 Wurcel AG, Anderson JE, Chui KKH, *et al.* Increasing Infectious Endocarditis Admissions Among Young People Who Inject Drugs. *Open Forum Infect Dis* 2016;**3**:ofw157. doi:10.1093/ofid/ofw157

19 Barocas JA, Eftekhari Yazdi G, Savinkina A, *et al.* Long-term Infective Endocarditis Mortality Associated With Injection Opioid Use in the United States: A Modeling Study. *Clin Infect Dis* 2020;**Online ahead of print.**:ciaa1346. doi:10.1093/cid/ciaa1346

20 Wright A, Otome O, Harvey C, *et al.* The Current Epidemiology of Injecting Drug Use-Associated Infective Endocarditis in Victoria, Australia in the Midst of Increasing Crystal Methamphetamine Use. *Heart Lung Circ* 2018;**27**:484–8. doi:10.1016/j.hlc.2017.03.166

21 Asgeirsson H, Thalme A, Weiland O. Low mortality but increasing incidence of Staphylococcus aureus endocarditis in people who inject drugs. *Medicine (Baltimore)* 2016;**95**. doi:10.1097/MD.0000000000005617

22 Gordon RJ, Lowy FD. Bacterial Infections in Drug Users. *N Engl J Med* 2005;**353**:1945–54. doi:10.1056/NEJMra042823

23 Dwyer R, Topp L, Maher L, *et al.* Prevalences and correlates of non-viral injecting-related injuries and diseases in a convenience sample of Australian injecting drug users. *Drug Alcohol Depend* 2009;**100**:9–16. doi:10.1016/j.drugalcdep.2008.08.016

24 Hope VD, Hickman M, Parry JV, *et al.* Factors associated with recent symptoms of an injection site infection or injury among people who inject drugs in three English cities. *Int J Drug Policy* 2014;**25**:303–7. doi:10.1016/j.drugpo.2013.11.012

25 Phillips KT, Stein MD. Risk Practices Associated with Bacterial Infections among Injection Drug Users in Denver, Colorado. *Am J Drug Alcohol Abuse* 2010;**36**:92–7. doi:10.3109/00952991003592311

For peer review only

26 Roux P, Donadille C, Magen C, *et al.* Implementation and evaluation of an educational intervention for safer injection in people who inject drugs in Europe: a multi-country mixed-methods study. *Int J Drug Policy* 2021;**87**:102992. doi:10.1016/j.drugpo.2020.102992

27 Stein MD, Phillips KT, Herman DS, *et al.* Skin-cleaning among hospitalized people who inject drugs: a randomized controlled trial. *Addiction* 2021;116(5):1122-1130. doi:https://doi.org/10.1111/add.15236

28 Bourgois P, Holmes SM, Sue K, *et al.* Structural Vulnerability: Operationalizing the Concept to Address Health Disparities in Clinical Care. *Acad Med* 2017;**92**:299–307. doi:10.1097/ACM.0000000000001294

29 Dasgupta N, Beletsky L, Ciccarone D. Opioid Crisis: No Easy Fix to Its Social and Economic Determinants. *Am J Public Health* 2017;**108**:182–6. doi:10.2105/AJPH.2017.304187

30 Okechukwu C, Davison K, Emmons K. Changing Health Behaviors in a Social Context. In: Berkman LF, Kawachi I, Glymour MM, eds. *Social Epidemiology*. Oxford University Press 2014. 365–95. doi:10.1093/med/9780195377903.003.0010

31 Glass TA, McAtee MJ. Behavioral science at the crossroads in public health: Extending horizons, envisioning the future. *Soc Sci Med* 2006;**62**:1650–71. doi:10.1016/j.socscimed.2005.08.044

32 Luchenski S, Maguire N, Aldridge RW, *et al.* What works in inclusion health: overview of effective interventions for marginalised and excluded populations. *The Lancet* 2018;**391**:266–80. doi:10.1016/S0140-6736(17)31959-1

33 Strathdee SA, Hallett TB, Bobrova N, *et al.* HIV and risk environment for injecting drug users: the past, present, and future. *Lancet Lond Engl* 2010;**376**:268–84. doi:10.1016/S0140-6736(10)60743-X

34 Rhodes T. The 'risk environment': a framework for understanding and reducing drug-related harm. *Int J Drug Policy* 2002;**13**:85–94. doi:10.1016/S0955-3959(02)00007-5

35 Rhodes T. Risk environments and drug harms: A social science for harm reduction approach. *Int J Drug Policy* 2009;**20**:193–201. doi:10.1016/j.drugpo.2008.10.003

36 Rhodes T, Singer M, Bourgois P, *et al.* The social structural production of HIV risk among injecting drug users. *Soc Sci Med* 2005;**61**:1026–44. doi:10.1016/j.socscimed.2004.12.024

37 Collins AB, Boyd J, Cooper HLF, *et al.* The intersectional risk environment of people who use drugs. *Soc Sci Med* 2019;**234**:112384. doi:10.1016/j.socscimed.2019.112384

38 Rhodes T, Simic M. Transition and the HIV risk environment. *BMJ* 2005;**331**:220–3.

39 Harris M, Rhodes T. Hepatitis C treatment access and uptake for people who inject drugs: a review mapping the role of social factors. *Harm Reduct J* 2013;**10**:7. doi:10.1186/1477-7517-10-7

40 McLean K. "There's nothing here": Deindustrialization as risk environment for overdose. *Int J Drug Policy* 2016;**29**:19–26. doi:10.1016/j.drugpo.2016.01.009

For peer review only - http://bmjopen.bmj.com/site/about/guidelines.xhtml

41 McNeil R, Small W. 'Safer environment interventions': A qualitative synthesis of the experiences and perceptions of people who inject drugs. *Soc Sci Med* 2014;**106**:151–8. doi:10.1016/j.socscimed.2014.01.051

42 Harris M, Scott J, Hope V, *et al.* Navigating environmental constraints to injection preparation: the use of saliva and other alternatives to sterile water among unstably housed PWID in London. *Harm Reduct J* 2020;**17**:24. doi:10.1186/s12954-020-00369-0

43 Unsigned. Heart Infection Projected to Kill One in Five People Who Inject Opioids by 2030. *Filter* 2020.https://filtermag.org/opioids-endocarditis-deaths/ (accessed 9 Dec 2020).

44 Harris M. Normalised pain and severe health care delay among people who inject drugs in London: Adapting cultural safety principles to promote care. *Soc Sci Med* 2020;**260**:113183. doi:10.1016/j.socscimed.2020.113183

45 Perlman DC, Jordan AE. The Syndemic of Opioid Misuse, Overdose, HCV, and HIV: Structural-Level Causes and Interventions. *Curr HIV/AIDS Rep* 2018;**15**:96–112. doi:10.1007/s11904-018-0390-3

46 Blankenship KM, Friedman SR, Dworkin S, *et al.* Structural Interventions: Concepts, Challenges and Opportunities for Research. *J Urban Health* 2006;**83**:59–72. doi:10.1007/s11524-005-9007-4

47 Moher D, Shamseer L, Clarke M, *et al.* Preferred reporting items for systematic review and meta-analysis protocols (PRISMA-P) 2015 statement. *Syst Rev* 2015;**4**:1. doi:10.1186/2046-4053-4-1

48 Shamseer L, Moher D, Clarke M, *et al.* Preferred reporting items for systematic review and meta-analysis protocols (PRISMA-P) 2015: elaboration and explanation. *BMJ* 2015;**349**:g7647. doi:10.1136/bmj.g7647

49 Moola S, Munn Z, Sears K, *et al.* Conducting systematic reviews of association (etiology): The Joanna Briggs Institute's approach. *JBI Evid Implement* 2015;**13**:163–9. doi:10.1097/XEB.0000000000000064

50 Pluye P, Hong QN. Combining the Power of Stories and the Power of Numbers: Mixed Methods Research and Mixed Studies Reviews. *Annu Rev Public Health* 2014;**35**:29–45. doi:10.1146/annurev-publhealth-032013-182440

51 Pluye P, Hong QN, Bush PL, *et al.* Opening-up the definition of systematic literature review: the plurality of worldviews, methodologies and methods for reviews and syntheses. *J Clin Epidemiol* 2016;**73**:2–5. doi:10.1016/j.jclinepi.2015.08.033

52 Tricco AC, Antony J, Soobiah C, *et al.* Knowledge synthesis methods for integrating qualitative and quantitative data: a scoping review reveals poor operationalization of the methodological steps. *J Clin Epidemiol* 2016;**73**:29–35. doi:10.1016/j.jclinepi.2015.12.011

53 Dixon-Woods M, Agarwal S, Jones D, *et al.* Synthesising qualitative and quantitative evidence: A review of possible methods. *J Health Serv Res Policy* 2005;**10**:45–53. doi:10.1177/135581960501000110

For peer review only - http://bmjopen.bmj.com/site/about/guidelines.xhtml

54 Heyvaert M, Hannes K, Onghena P. *Using Mixed Methods Research Synthesis for Literature Reviews: The Mixed Methods Research Synthesis Approach*. SAGE Publications 2016.

55 Sandelowski M, Leeman J, Knafl K, *et al.* Text-in-context: a method for extracting findings in mixed-methods mixed research synthesis studies. *J Adv Nurs* 2013;**69**:1428–37. doi:10.1111/jan.12000

56 Rhodes T, Wagner K, Strathdee SA, *et al.* Structural Violence and Structural Vulnerability Within the Risk Environment: Theoretical and Methodological Perspectives for a Social Epidemiology of HIV Risk Among Injection Drug Users and Sex Workers. In: O'Campo P, Dunn JR, eds. *Rethinking Social Epidemiology: Towards a Science of Change*. Dordrecht: : Springer Netherlands 2012. 205–30. doi:10.1007/978-94-007-2138-8_10

57 Lambdin BH, Bluthenthal RN, Zibbell JE, *et al.* Associations between perceived illicit fentanyl use and infectious disease risks among people who inject drugs. *Int J Drug Policy* 2019;**74**:299–304. doi:10.1016/j.drugpo.2019.10.004

58 Linton SL, Celentano DD, Kirk GD, *et al.* The longitudinal association between homelessness, injection drug use, and injection-related risk behavior among persons with a history of injection drug use in Baltimore, MD. *Drug Alcohol Depend* 2013;**132**:457–65. doi:10.1016/j.drugalcdep.2013.03.009

59 Genberg BL, Gange SJ, Go VF, *et al.* Trajectories of Injection Drug Use Over 20 Years (1988–2008) in Baltimore, Maryland. *Am J Epidemiol* 2011;**173**:829–36. doi:10.1093/aje/kwq441

60 Bundle N, Bubba L, Coelho J, *et al.* Ongoing outbreak of invasive and non-invasive disease due to group A Streptococcus (GAS) type emm66 among homeless and people who inject drugs in England and Wales, January to December 2016. *Eurosurveillance* 2017;**22**. doi:10.2807/1560-7917.ES.2017.22.3.30446

61 Parikh MP, Octaria R, Kainer MA. Methicillin-Resistant Staphylococcus aureus Bloodstream Infections and Injection Drug Use, Tennessee, USA, 2015–2017 - Volume 26, Number 3—March 2020 - Emerging Infectious Diseases journal - CDC. *Emerg Infect Dis* 2020;**26**:446–53. doi:10.3201/eid2603.191408

62 Greenhalgh T, Peacock R. Effectiveness and efficiency of search methods in systematic reviews of complex evidence: audit of primary sources. *BMJ* 2005;**331**:1064–5. doi:10.1136/bmj.38636.593461.68

63 Krüsi A, Wood E, Montaner J, *et al.* Social and structural determinants of HAART access and adherence among injection drug users. *Int J Drug Policy* 2010;**21**:4–9. doi:10.1016/j.drugpo.2009.08.003

64 Milloy M-J, Marshall B, Kerr T, *et al.* Social and structural factors associated with HIV disease progression among illicit drug users: A systematic review. *AIDS Lond Engl* 2012;**26**:1049–63. doi:10.1097/QAD.0b013e32835221cc

For peer review only - http://bmjopen.bmj.com/site/about/guidelines.xhtml

65 Gicquelais RE, Werb D, Marks C, *et al.* Prevalence and Correlates of Providing and Receiving Assistance With the Transition to Injection Drug Use. *Epidemiol Rev* 2020;**42**:4–18. doi:10.1093/epirev/mxaa008

66 Alvarez E, Joshi S, Lokker C, *et al.* Health programmes and services addressing the prevention and management of infectious diseases in persons who inject drugs in Canada: a systematic integrative review protocol. *BMJ Open* 2020;**10(8):** e035188. doi:10.1136/bmjopen-2019-035188

67 Kudrina I, Puzhko S, Filion KB, *et al.* Effectiveness of Interventions for Prevention of Common Infections in People Who Use Opioids: A Protocol for a Systematic Review of Systematic Reviews (Preprint). *ResearchSquare* Published Online First: 2020. doi:10.21203/rs.3.rs-114546/v1

68 Shah M, Wong R, Ball L, *et al.* Risk factors of infective endocarditis in persons who inject drugs. *Harm Reduct J* 2020;**17**:35. doi:10.1186/s12954-020-00378-z

69 Silverman M, Slater J, Jandoc R, *et al.* Hydromorphone and the risk of infective endocarditis among people who inject drugs: a population-based, retrospective cohort study. *Lancet Infect Dis* 2020;**20(4)**:487-497. doi:10.1016/S1473-3099(19)30705-4

70 Ciccarone D, Unick GJ, Cohen J, *et al.* Nationwide Increase in Hospitalizations for Heroin-related Soft Tissue Infections: Associations with Structural Market Conditions. *Drug Alcohol Depend* 2016;**163**:126–33. doi:10.1016/j.drugalcdep.2016.04.009

71 Harris M, Scott J, Wright T, *et al.* Injecting-related health harms and overuse of acidifiers among people who inject heroin and crack cocaine in London: a mixed-methods study. *Harm Reduct J* 2019;**16**:60. doi:10.1186/s12954-019-0330-6

72 Yeung A, Weir A, Austin H, *et al.* Assessing the impact of a temporary class drug order on ethylphenidate-related infections among people who inject drugs in Lothian, Scotland: an interrupted time–series analysis. *Addiction* 2017;**112**:1799–807. doi:https://doi.org/10.1111/add.13898

73 Trayner KMA, McAuley A, Palmateer NE, *et al.* Increased risk of HIV and other drug-related harms associated with injecting in public places: national bio-behavioural survey of people who inject drugs. *Int J Drug Policy* 2020;**77**:102663. doi:10.1016/j.drugpo.2020.102663

74 Hong QN, Fàbregues S, Bartlett G, *et al.* The Mixed Methods Appraisal Tool (MMAT) version 2018 for information professionals and researchers. *Educ Inf* 2018;**34**:285–91. doi:10.3233/EFI-180221

75 Hong QN, Pluye P. A Conceptual Framework for Critical Appraisal in Systematic Mixed Studies Reviews. *J Mix Methods Res* 2019;**13**:446–60. doi:10.1177/1558689818770058

76 Hsieh H-F, Shannon SE. Three Approaches to Qualitative Content Analysis. *Qual Health Res* 2005;**15**:1277–88. doi:10.1177/1049732305276687

77 Deeks JJ, Higgins JP, Altman DG. Analysing data and undertaking meta-analyses. In: *Cochrane Handbook for Systematic Reviews of Interventions*. John Wiley & Sons, Ltd 2019. 241–84. doi:10.1002/9781119536604.ch10

For peer review only - http://bmjopen.bmj.com/site/about/guidelines.xhtml

78 Platt L, Grenfell P, Meiksin R, *et al.* Associations between sex work laws and sex workers' health: A systematic review and meta-analysis of quantitative and qualitative studies. *PLOS Med* 2018;**15**:e1002680. doi:10.1371/journal.pmed.1002680

79 Bonn M, Palayew A, Bartlett S, *et al.* Addressing the Syndemic of HIV, Hepatitis C, Overdose, and COVID-19 Among People Who Use Drugs: The Potential Roles for Decriminalization and Safe Supply. *J Stud Alcohol Drugs* 2020;**81**:556–60. doi:10.15288/jsad.2020.81.556

80 Brothers TD, Bonn M. Patient-centred care in opioid agonist treatment could improve outcomes. *Can Med Assoc J* 2019;**191**:E460–1. doi:10.1503/cmaj.190430

81 Bonn M, Touesnard N, Cheng B, *et al.* Barriers and facilitators to accessing and providing a safe supply of drugs during COVID-19 and beyond: a scoping review. *Submitted*

82 Brothers TD, Fraser J, MacAdam E, *et al.* Implementation and evaluation of a novel, unofficial, trainee-organized hospital addiction medicine consultation service. *Subst Abuse* 2020;**Online ahead of print**:1–8. doi:10.1080/08897077.2020.1856291

83 Brothers TD, Webster D. Multidisciplinary infective endocarditis care teams should address substance use disorders and harm reduction services. *Open Heart* 2018;**4**. doi:10.1136/openhrt-2017-000699

84 Brothers TD, Fraser J, Webster D. Caring for people who inject drugs when they are admitted to hospital. *CMAJ Can Med Assoc J* 2021;**193**:E423–4. doi:doi: 10.1503/cmaj.202124

85 Friedman SR, Krawczyk N, Perlman DC, *et al.* The Opioid/Overdose Crisis as a Dialectics of Pain, Despair, and One-Sided Struggle. *Front Public Health* 2020;**8**. doi:10.3389/fpubh.2020.540423

86 McCall J, Pauly B. Sowing a Seed of Safety: Providing Culturally Safe Care in Acute Care Settings for People who use Drugs. *J Ment Health Addict Nurs* 2019;**3**:e1–7. doi:10.22374/jmhan.v3i1.33

87 Lennox R, Martin L, Brimner C, *et al.* Hospital policy as a harm reduction intervention for people who use drugs. *Int J Drug Policy* 2021;**97**:103324. doi:10.1016/j.drugpo.2021.103324

88 Turner S. Numerous outbreaks amongst homeless and injection drug-using populations raise concerns of an evolving syndemic in London, Canada. *Epidemiol Infect* 2020;**148**:e160. doi:10.1017/S0950268820001260

89 Tiako MJN, Mszar R, Brooks C, *et al.* Cardiac surgeons' treatment approaches for infective endocarditis based on patients' substance use history. *Semin Thorac Cardiovasc Surg* Published Online First: 3 December 2020. doi:10.1053/j.semtcvs.2020.11.031

90 An KR, Luc JGY, Tam DY, *et al.* Infective endocarditis secondary to injection drug use: A survey of Canadian cardiac surgeons. *Ann Thorac Surg* 2020;**0**. doi:10.1016/j.athoracsur.2020.12.003

For peer review only

91 Buchanan NE, Lennox RB, Whitlock R, *et al.* Making the Cut: Perspectives on the Surgical Management of Infective Endocarditis Among People Who Use Intravenous Drugs. *Can J Cardiol* 2019;**35**:1416–8. doi:10.1016/j.cjca.2019.05.016

92 Harris M, Rhodes T. Venous access and care: harnessing pragmatics in harm reduction for people who inject drugs. *Addiction* 2012;**107**:1090–6.

93 Friedman SR, Neaigus A, Jose B, *et al.* Networks and HIV risk: an introduction to social network analysis for harm reductionists. *Int J Drug Policy* 1998;**9**:461–9. doi:10.1016/S0955-3959(98)00062-0

94 Al-Rawahi GN, Schreader AG, Porter SD, *et al.* Methicillin-resistant Staphylococcus aureus nasal carriage among injection drug users: six years later. *J Clin Microbiol* 2008;**46**:477–9. doi:10.1128/JCM.01596-07

95 Kimura AC, Higa JI, Levin RM, *et al.* Outbreak of Necrotizing Fasciitis Due to Clostridium sordellii among Black-Tar Heroin Users. *Clin Infect Dis* 2004;**38**:e87–91. doi:10.1086/383471

96 Price EP, Seymour ML, Sarovich DS, *et al.* Molecular epidemiologic investigation of an anthrax outbreak among heroin users, Europe. *Emerg Infect Dis* 2012;**18**:1307–13. doi:10.3201/eid1808.111343

97 Pantell MS, Adler-Milstein J, Wang MD, *et al.* A call for social informatics. *J Am Med Inform Assoc* 2020;**27**:1798–801. doi:10.1093/jamia/ocaa175

For peer review only

For peer review only - http://bmjopen.bmj.com/site/about/guidelines.xhtml

729 **Table 1. A model of the risk environment tabular summary**

	**Microenvironmental**	**Macroenvironmental**
Social environment		
Physical environment		
Economic environment		
Policy environment		

730 Modified from Rhodes, 2002.[34]
731

For peer review only

For peer review only - http://bmjopen.bmj.com/site/about/guidelines.xhtml

Figure captions

Figure 1. Illustrative schematic of a potential risk environment for injecting-related bacterial and fungal infections, as it may structure or create infection risk during the process of drug injection/consumption. Environmental factors, which are external to individuals, interact to influence individual-level factors and health behaviours across stages of a potential pathway: drug acquisition; drug preparation; drug injection; treatment of superficial injection-site bacterial and fungal infections (e.g. in primary care or emergency departments); treatment of severe/invasive bacterial and fungal infections (e.g. in hospital for intravenous antibiotics and/or surgery); and health outcomes after severe/invasive bacterial and fungal infections (e.g. disability, death).

Figure 2. Illustrative schematic of a potential risk environment for injecting-related bacterial and fungal infections, as it may structure or create infection risk during the process of recognition and adequate treatment of superficial injection-site infections. Environmental factors, which are external to individuals, interact to influence individual-level factors and health behaviours across stages of a potential pathway: drug acquisition; drug preparation; drug injection; treatment of superficial injection-site bacterial and fungal infections (e.g. in primary care or emergency departments); treatment of severe/invasive bacterial and fungal infections (e.g. in hospital for intravenous antibiotics and/or surgery); and health outcomes after severe/invasive bacterial and fungal infections (e.g. disability, death).

For peer review only

For peer review only

For peer review only - http://bmjopen.bmj.com/site/about/guidelines.xhtml

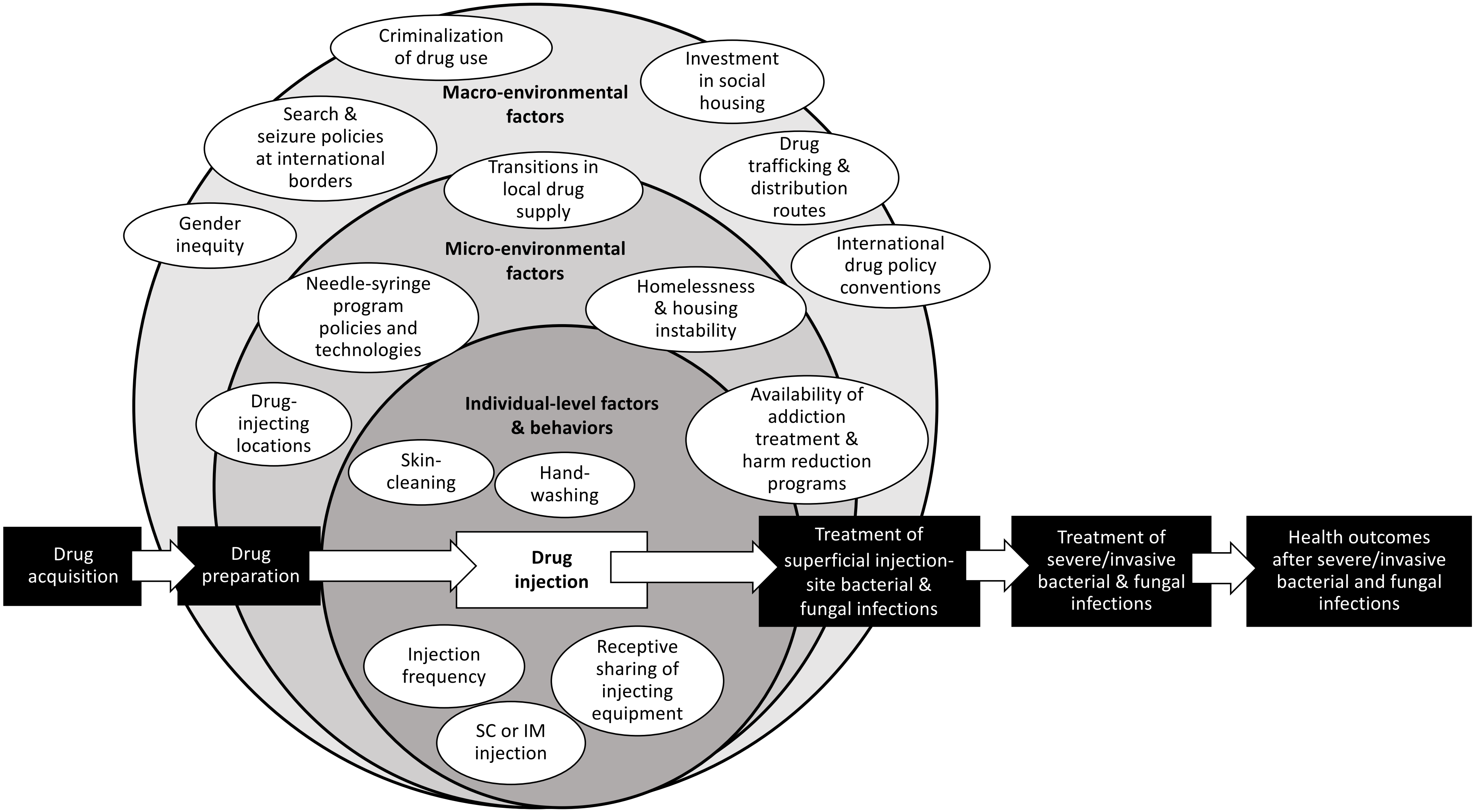
Criminalization of drug use
Investment in social housing
Macro-environmental factors
Search & seizure policies at international borders
Drug trafficking & distribution routes
Transitions in local drug supply
Gender inequity
International drug policy conventions
Micro-environmental factors
Needle-syringe program policies and technologies
Homelessness & housing instability
Drug-injecting locations
Individual-level factors & behaviors
Availability of addiction treatment & harm reduction programs
Skin-cleaning
Hand-washing
Drug acquisition
Drug preparation
Drug injection
Treatment of superficial injection-site bacterial & fungal infections
Treatment of severe/invasive bacterial & fungal infections
Health outcomes after severe/invasive bacterial and fungal infections
Injection frequency
Receptive sharing of injecting equipment
SC or IM injection

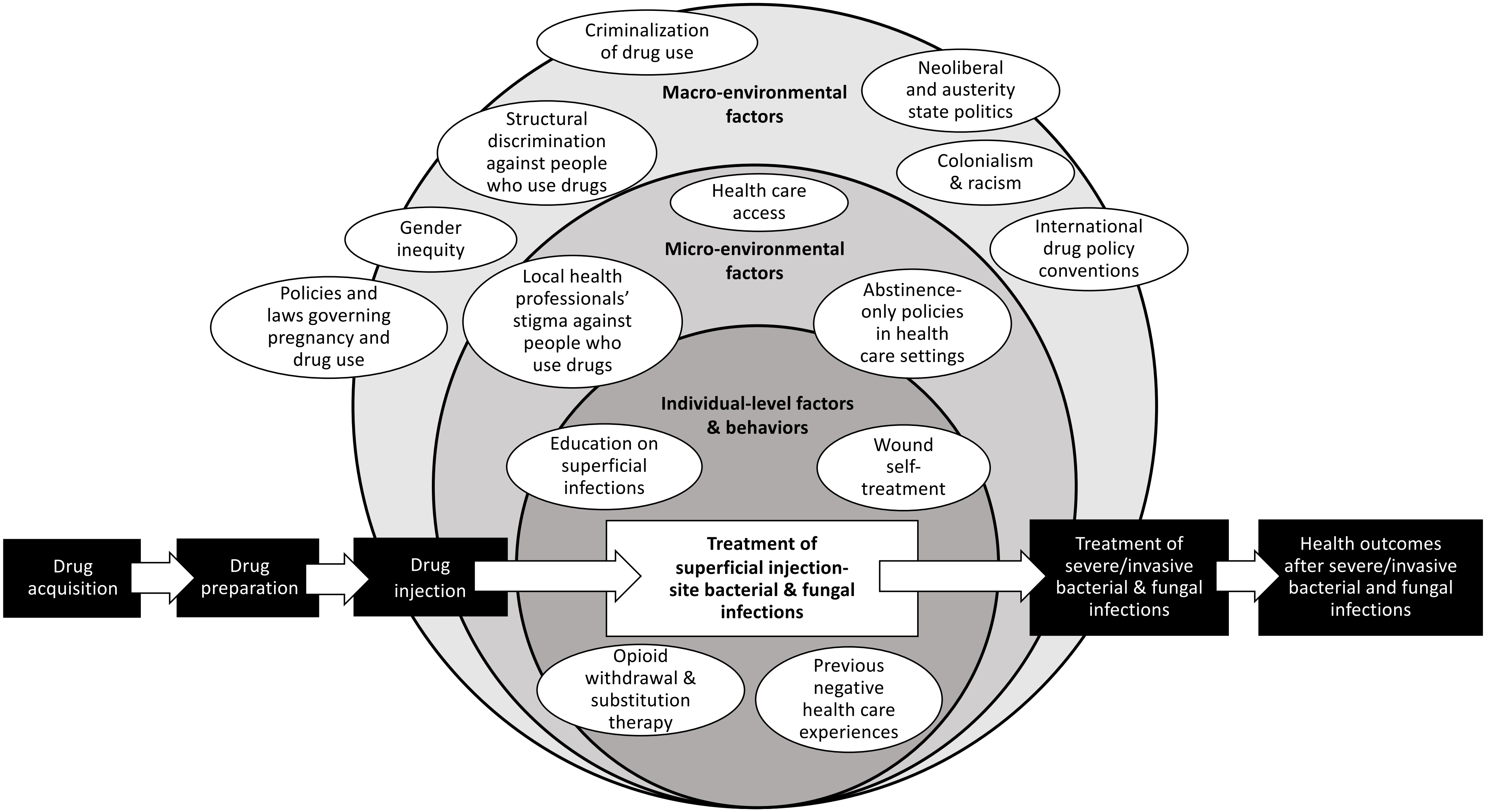
Criminalization of drug use
Neoliberal and austerity state politics
Macro-environmental factors
Structural discrimination against people who use drugs
Colonialism & racism
Health care access
Gender inequity
International drug policy conventions
Micro-environmental factors
Policies and laws governing pregnancy and drug use
Local health professionals' stigma against people who use drugs
Abstinence-only policies in health care settings
Individual-level factors & behaviors
Education on superficial infections
Wound self-treatment
Drug acquisition
Drug preparation
Drug injection
Treatment of superficial injection-site bacterial & fungal infections
Treatment of severe/invasive bacterial & fungal infections
Health outcomes after severe/invasive bacterial and fungal infections
Opioid withdrawal & substitution therapy
Previous negative health care experiences

Supplementary materials for the article:

Social and structural determinants of injecting-related bacterial and fungal infections among people who inject drugs: protocol for a mixed studies systematic review

Thomas D. Brothers[1,2]; Dan Lewer[1]; Matthew Bonn[3]; Duncan Webster[2,4]; Magdalena Harris[5]

[1]UCL Collaborative Centre for Inclusion Health, Institute of Epidemiology & Health Care, University College London (UCL), London, UK
[2]Department of Medicine, Dalhousie University, Halifax, Canada
[3]Canadian Association of People who Use Drugs (CAPUD), Dartmouth, Canada
[4]Division of Infectious Diseases, Saint John Regional Hospital, Saint John, Canada
[5]Department of Public Health, Environments and Society, London School of Hygiene and Topical Medicine (LSHTM), London, UK

This material supplements, but does not replace, the peer-reviewed article in BMJ Open

For peer review only

Supplementary appendix Table 1. Pilot search strategy developed in PubMed on February 1, 2021 (1,752 results)

Concepts	PubMed
People who inject drugs, or drug preparation and injection	(“Substance-Related Disorders”[MeSH] OR
	“Substance Abuse, Intravenous”[MeSH] OR
	“Drug Users”[MeSH] OR
	“Needle Sharing”[MeSH] OR
	“people who inject drugs”[tiab] OR “persons who inject drugs”[tiab] OR PWID[tiab] OR
	“people who use drugs”[tiab] OR “persons who use drugs”[tiab] OR PWUD[tiab] OR
	“injection drug”[tiab] OR IDU[tiab] OR
	“intravenous drug”[tiab] OR IVDU[tiab] OR
	“drug abuse”[tiab] OR
	“illicit drugs”[MeSH] OR “illicit drug”[tiab] OR “illegal drug”[tiab] OR
	“Heroin”[MeSH] OR Heroin[tiab] OR
	“Heroin Dependence”[MeSH] OR
	“Opiate use disorder”[tiab] OR “opioid use disorder”[tiab] OR “opiate dependence”[tiab] OR “opioid dependence”[tiab] OR “opiate abuse”[tiab] OR “opioid abuse”[tiab] OR “substance use disorder”[tiab] OR “substance abuse”[tiab] OR
	“Cocaine”[MeSH] OR cocaine[tiab] OR
	“Crack Cocaine”[MeSH] OR
	“groin injecting”[tiab] OR “femoral injecting”[tiab] OR
	“Harm Reduction”[MeSH] OR “harm reduction”[tiab] OR
	“Needle-Exchange Programs”[MeSH] OR

For peer review only

	“needle exchange”[tiab] OR “syringe exchange”[tiab] OR “syringe services”[tiab] OR “syringe program”[tiab] OR
	acidifier*[tiab] OR
	“Opiate Substitution Treatment”[MeSH] OR “opiate substitution”[tiab] OR “opiate agonist”[tiab] OR “opioid substitution”[tiab] OR “opioid agonist”[tiab] OR
	“Medications for opioid use disorder”[tiab] OR MOUD[tiab] OR
	Methadone[tiab] OR
	Buprenorphine[tiab] OR
	Hydromorphone[tiab] OR morphine[tiab] or fentanyl[tiab])
Injecting-related infections	AND
	(“injection-related infections”[tiab] OR “injection-related infection”[tiab] OR
	“bacterial infection”[tiab] OR “bacterial infections”[tiab] OR
	Bacteremia[MeSH] OR bacteremia[tiab] OR
	Fungemia[MeSH] OR
	Cellulitis[MeSH] OR cellulitis[tiab] OR
	Abscess[MeSH] OR abscess*[tiab] OR
	“skin infection”[tiab] OR “skin infections”[tiab] OR
	“skin and soft tissue”[tiab] OR SSTI*[tiab] OR
	Endocarditis[MeSH] OR endocarditis[tiab] OR
	Bone Diseases, Infectious[MeSH] OR
	Osteomyelitis[MeSH] OR Osteomyelitis[tiab] OR
	“septic arthritis”[tiab] OR
	Central Nervous System Infections[MeSH] OR

For peer review only

	Gram-Positive Bacterial Infections[MeSH] OR
	Candidiasis[MeSH])
Social and structural determinants, or risk environment	AND
	("risk factor"[tiab] OR “risk factors”[tiab] OR
	correlate*[tiab] OR
	determinant*[tiab] OR
	environment*[tiab] OR
	“social factors”[tiab] or “structural factors”[tiab] OR
	Cohort*[tiab] OR
	Longitudinal[tiab] OR
	Prospective[tiab] OR retrospective[tiab] OR
	Randomized[tiab] OR randomised[tiab] OR
	Comparative[tiab] OR
	Case-control[tiab] OR
	Time-series[tiab] OR
	Survey*[tiab] OR
	Epidemiolog*[tiab] OR
	Qualitative[tiab] OR
	Interview[tiab] OR
	Ethnograph*[tiab] OR
	Mixed-methods[tiab] OR “mixed methods”[tiab] OR
	gender[tiab] OR
	homeless*[tiab] OR

For peer review only

race[tiab] OR racism[tiab] OR

incarcerat*[tiab] OR prison*[tiab] OR criminal*[tiab] OR

stigma*[tiab] OR discrimination[tiab] OR exclusion[tiab])

NOT (“case report”[Title]) NOT (“case series”[Title])

Filter: 2000-Present

For peer review only

Reporting checklist for protocol of a systematic review.

Based on the PRISMA-P guidelines.

Instructions to authors

Complete this checklist by entering the page numbers from your manuscript where readers will find each of the items listed below.

Your article may not currently address all the items on the checklist. Please modify your text to include the missing information. If you are certain that an item does not apply, please write "n/a" and provide a short explanation.

Upload your completed checklist as an extra file when you submit to a journal.

In your methods section, say that you used the PRISMA-Preporting guidelines, and cite them as:

Moher D, Shamseer L, Clarke M, Ghersi D, Liberati A, Petticrew M, Shekelle P, Stewart LA. Preferred Reporting Items for Systematic Review and Meta-Analysis Protocols (PRISMA-P) 2015 statement. Syst Rev. 2015;4(1):1.

		Reporting Item	Page Number
**Title**			
Identification	#1a	Identify the report as a protocol of a systematic review	1
Update	#1b	If the protocol is for an update of a previous systematic review, identify as such	N/A
**Registration**			
	#2	If registered, provide the name of the registry (such as PROSPERO) and registration number	3 & 7
**Authors**			
Contact	#3a	Provide name, institutional affiliation, e-mail address of all protocol authors; provide physical mailing address of corresponding author	1
Contribution	#3b	Describe contributions of protocol authors and identify the guarantor of the review	17
**Amendments**			

For peer review only

	#4	If the protocol represents an amendment of a previously completed or published protocol, identify as such and list changes; otherwise, state plan for documenting important protocol amendments	N/A
**Support**			
Sources	#5a	Indicate sources of financial or other support for the review	17
Sponsor	#5b	Provide name for the review funder and / or sponsor	N/A
Role of sponsor or funder	#5c	Describe roles of funder(s), sponsor(s), and / or institution(s), if any, in developing the protocol	17
**Introduction**			
Rationale	#6	Describe the rationale for the review in the context of what is already known	5-6
Objectives	#7	Provide an explicit statement of the question(s) the review will address with reference to participants, interventions, comparators, and outcomes (PICO)	6
**Methods**			
Eligibility criteria	#8	Specify the study characteristics (such as PICO, study design, setting, time frame) and report characteristics (such as years considered, language, publication status) to be used as criteria for eligibility for the review	8
Information sources	#9	Describe all intended information sources (such as electronic databases, contact with study authors, trial registers or other grey literature sources) with planned dates of coverage	10-11
Search strategy	#10	Present draft of search strategy to be used for at least one electronic database, including planned limits, such that it could be repeated	10-11
Study records - data management	#11a	Describe the mechanism(s) that will be used to manage records and data throughout the review	12
Study records - selection process	#11b	State the process that will be used for selecting studies (such as two independent reviewers) through each phase of the review (that is, screening, eligibility and inclusion in meta-analysis)	12
Study records - data collection process	#11c	Describe planned method of extracting data from reports (such as piloting forms, done independently, in duplicate), any processes for	12

For peer review only

		obtaining and confirming data from investigators	
Data items	#12	List and define all variables for which data will be sought (such as PICO items, funding sources), any pre-planned data assumptions and simplifications	12
Outcomes and prioritization	#13	List and define all outcomes for which data will be sought, including prioritization of main and additional outcomes, with rationale	12
Risk of bias in individual studies	#14	Describe anticipated methods for assessing risk of bias of individual studies, including whether this will be done at the outcome or study level, or both; state how this information will be used in data synthesis	13
Data synthesis	#15a	Describe criteria under which study data will be quantitatively synthesised	13
Data synthesis	#15b	If data are appropriate for quantitative synthesis, describe planned summary measures, methods of handling data and methods of combining data from studies, including any planned exploration of consistency (such as I2, Kendall's τ)	N/A
Data synthesis	#15c	Describe any proposed additional analyses (such as sensitivity or subgroup analyses, meta-regression)	N/A
Data synthesis	#15d	If quantitative synthesis is not appropriate, describe the type of summary planned	13
Meta-bias(es)	#16	Specify any planned assessment of meta-bias(es) (such as publication bias across studies, selective reporting within studies)	N/A
Confidence in cumulative evidence	#17	Describe how the strength of the body of evidence will be assessed (such as GRADE)	13

The PRISMA-P checklist is distributed under the terms of the Creative Commons Attribution License CC-BY 4.0. This checklist was completed on 27. January 2021 using https://www.goodreports.org/, a tool made by the EQUATOR Network in collaboration with Penelope.ai

For peer review only
